# Homometallic 2D Cd^2+^ and Heterometallic 3D Cd^2+^/Ca^2+^, Cd^2+^/Sr^2+^ Metal–Organic Frameworks Based on an Angular Tetracarboxylic Ligand

**DOI:** 10.3390/ma18204647

**Published:** 2025-10-10

**Authors:** Rafail P. Machattos, Nikos Panagiotou, Vasiliki I. Karagianni, Manolis J. Manos, Eleni E. Moushi, Anastasios J. Tasiopoulos

**Affiliations:** 1 Department of Chemistry, University of Cyprus, 1678 Nicosia, Cyprus; rmacha01@ucy.ac.cy (R.P.M.); panagiotou.nikos@ucy.ac.cy (N.P.); 2 Department of Chemistry, University of Ioannina, 45110 Ioannina, Greece; v.karagianni@uoi.gr (V.I.K.); emanos@uoi.gr (M.J.M.); 3 Department of Life Sciences, European University Cyprus, 2404 Nicosia, Cyprus

**Keywords:** cadmium, alkaline earths, metal–organic frameworks, angular tetracarboxylic ligands, crystal structures, gas sorption properties

## Abstract

This study reports on the synthesis, structural characterization and gas sorption studies of a homometallic 2D Cd^2+^ MOF and two heterometallic 3D Cd^2+^/Ca^2+^ and Cd^2+^/Sr^2+^ -MOFs based on the angular tetracarboxylic ligand 3,3′,4,4′-sulfonyltetracarboxylic acid (H_4_STBA). The homometallic 2D Cd^2+^ MOF with the formula [NH_2_(CH_3_)_2_]^+^_2_[Cd(STBA)]^2−^_n_·nDMF·1.5nH_2_O—(**1**)_n_·nDMF·1.5nH_2_O was synthesized from the reaction of CdCl_2_·H_2_O and 3,3′,4,4′-diphthalic sulfonyl dianhydride (3,3′,4,4′-DPSDA) with stoichiometric ratio of 1:1.3 in DMF/H_2_O (5/2 mL) at 100 °C. The two heterometallic Cd^2+^/Ca^2+^ and Cd^2+^/Sr^2+^ compounds were prepared from analogous reactions to this afforded (**1**)_n_·nDMF·1.5nH_2_O with the difference that the reaction mixture also contained AE(NO_3_)_2_ (AE^2+^ = Ca^2+^ or Sr^2+^) and, in particular, from the reaction of AE(NO_3_)_2_, CdCl_2_·H_2_O and 3,3′,4,4′-DPSDA with stoichiometric ratio 1:1.1:1.4 in DMF/H_2_O (5/2 mL) at 100 °C. Notably, compounds [CdCa(STBA)(H_2_O)_2_]_n_·0.5nDMF—(**2**)_n_·0.5nDMF and [CdSr(STBA)(H_2_O)_2_]_n_·0.5nDMF—(**3**)_n_·0.5nDMF are the first heterometallic compounds M^n+^/AE^2+^ (M = any metal ion) reported containing ligand H_4_STBA. The structure of (**1**)_n_·nDMF·1.5nH_2_O comprises a 2D network based on helical 1D chain secondary building unit (SBU) [Cd^2+^(STBA)^4−^)]^2−^. The 2D sheets are linked through hydrogen bonding interactions, giving rise to a pseudo-3D structure. On the other hand, compounds (**2**)_n_·1.5nH_2_O and (**3**)_n_·1.5nH_2_O display 3D microporous structures consisting of a helical 1D chain SBU [Cd^2+^AE^2+^(STBA)^4−^)]. All three compounds contain rhombic channels along c axes. The three MOFs exhibit an appreciable thermal stability, up to 350–400 °C. Gas sorption measurements on activated materials (**2**)_n_ and (**3**)_n_ revealed moderate BET surface areas of 370 m^2^/g and 343 m^2^/g, respectively, along with CO_2_ uptake capacity of 2.58 mmol/g at 273 K.

## 1. Introduction

Metal–Organic Frameworks (MOFs) are considered a highly versatile and promising family of materials in the field of inorganic chemistry since their discovery in the late 20th century. MOFs consist of metal ions or clusters linked by organic bridging ligands, forming a porous, crystalline structure with high surface areas. Their unique structures arising from the variety of metal ions, organic ligands, functional groups and resulting network topologies enable tunability and customization [1,2,3,4,5], making them valuable for applications in fields of general interest, such as energy, environment, healthcare, food safety, etc. As a consequence, numerous MOFs have been synthesized and proposed for potential applications in the fields of gas storage/separation [6,7,8,9], catalysis [10,11,12], sensing [13,14,15,16], removal of pollutants from the environment [17,18,19,20], and water harvesting [21].

The construction of functional MOFs requires the judicious selection of not only the metal ions but also the organic linkers. Specifically, there are several factors regarding the identity of the appropriate carboxylate organic ligand, for the construction of MOFs, including the overall size of the molecule, rigidity/flexibility and the number and positions of the carboxylate groups [22,23,24,25,26,27,28]. One category of organic ligands widely used in MOF synthesis comprises semi-rigid, V-shaped multicarboxylic acid molecules that feature two benzoate or phthalate units connected through a central functional group or atom. Generally, the central group/atom can be an etheric -O- group [20,29,30,31,32,33,34,35,36,37,38,39], a thioetheric -S- atom [40], a secondary or tertiary amine; -(NH(R))- (R: H-, Me-, etc.) [41,42,43,44], a ketone group; -(C=O)- [31,45,46,47,48,49,50,51,52,53,54], a hexafluoroisopropylidene group; -C(CF_3_)_2_- [20,55,56,57,58,59,60,61,62,63,64], silane groups -(SiR′_2_)- (R′: Me-, Ph-, etc.) [65,66,67,68] or a sulfonyl group; -S(O)_2_- [69,70,71,72,73]. The flexibility of these di-/tetracrboxylate linkers stems from the sp^3^-hybridized central atom connecting the two benzoate or phthalate moieties in most of these linkers, which allows the rotation of the adjacent benzene rings around the central atom. Although there are numerous examples of MOFs based on V-shaped dicarboxylic linkers, including 4,4′-oxybisbenzoic acid and 4,4′-sulfonyl dibenzoic acid (amongst others), the corresponding examples with V-shaped tetracarboxylic ligands are significantly fewer. Among them, the MOF structures with diphthalic ligand derivatives are relatively rare, as revealed by a CCDC search, which returned around 310 examples for this family of ligands. This is possibly because of the incompatibility of the phthalic acid moiety with the common SBUs formed in MOFs. Another common issue with phthalic acid-containing ligands is that they tend to adopt various coordination modes with metal ions, restricting the ability to form targeted MOF structures with specific topological features. As a result, such ligands lead to several new structures with various network topologies, especially when they are used with metal ions possessing flexible coordination spheres (as lanthanide ions, alkaline earth ions, etc.). In addition, because of their multiple binding sites (the four carboxylate groups), they usually bind with several metal ions and, this way, they can stabilize heterometallic MOFs containing two or more different metal ions [36,52]. In recent years, heterometallic MOFs (HMOFs) have attracted significant attention, and have been employed in multiple applications, including H_2_ sorption and storage [74,75,76,77,78,79], light hydrocarbon sorption [80,81,82,83,84], catalysis [85,86,87,88,89,90], magnetism [91,92,93,94], and sensing [95,96].

Among the diphthalic ligands, 3,3′,4,4′-sulfonyltetracarboxylic acid (H_4_STBA) has a high bridging capability due to its four carboxylic groups, while the central –SO_2_– moiety can serve either as a binding site or as a functional group influencing the sorption performance of materials [69,71,97,98]. Therefore, the H_4_STBA ligand is a promising candidate for the synthesis of multidimensional coordination polymers, especially 3D porous frameworks. The reported coordination polymers containing the STBA^4−^ ligand are all based on individual metal ions with or without auxiliary ligands, and there are no M^n+^/AE^2+^ (M = any metal ion) heterometallic MOFs in the literature.

We herein report three new MOFs, one 2D Cd^2+^-MOF and two heterometallic microporous 3D Cd^2+^/Ca^2+^ and Cd^2+^/Sr^2+^ -MOFs with formulae [NH_2_(CH_3_)_2_]^+^_2_[Cd(STBA)]^2−^_n_·nDMF·1.5nH_2_O—(**1**)_n_·nDMF·1.5nH_2_O, [CdCa(STBA)(H_2_O)_2_]_n_·0.5nDMF—(**2**)_n_·0.5nDMF and [CdSr(STBA)(H_2_O)_2_]_n_·0.5nDMF—(**3**)_n_·0.5nDMF. The three compounds were synthesized by analogous synthetic routes involving the reaction of CdCl_2_·H_2_O with 3,3′,4,4′-diphthalic sulfonyl dianhydride (3,3′,4,4′-DPSDA), which was hydrolyzed in situ to afford the corresponding tetracarboxylic acid in DMF/H_2_O 5/2 mL at 100 °C in the presence or not (in the synthesis of (**1**)_n_) of Ca(NO_3_)_2_·4H_2_O (in the synthesis of (**2**)_n_) or Sr(NO_3_)_2_ (in the synthesis of (**3**)_n_). Compound (**1**)_n_·nDMF·1.5nH_2_O consists of a homometallic 1D helical Cd^2+^-chain SBU and exhibits a 2D structure, whereas compounds (**2**)_n_·0.5nDMF and (**3**)_n_·0.5nDMF feature a heterometallic 1D helical Cd^2+^AE^2+^-chain SBU and display 3D structures. In addition, all three MOFs exhibit appreciable thermal stability, retaining their structural integrity and crystallinity even at elevated temperatures (350–400 °C) as confirmed by thermogravimetric analysis (TGA) and variable temperature powder x-ray diffraction (VT-pXRD) measurements. CO_2_ uptake measurements on activated samples of (**2**)_n_ and (**3**)_n_ revealed type I isotherms, typical of microporous materials, with BET surface areas of 370 m^2^/g and 343 m^2^/g (Langmuir areas of 403 and 375 m^2^/g), respectively.

## 2. Materials and Methods

Details about the synthesis, stability studies, and the physicochemical characterization methods of compounds (**1**)_n_, (**2**)_n_, and (**3**)_n_ are provided in the Supplementary Materials.

## 3. Results and Discussion

### 3.1. Synthesis

In recent years, our group has explored the use of V-shaped dicarboxylic ligands in MOF chemistry, aiming to develop new functional materials. Several such ligands have been employed in these studies, comprising two benzoic acid units connected via a central linker, etheric -O- (H_2_OBA), hexafluoroisopropylidene, -C(CF_3_)_2_- (H_2_HFPBBA), carbonyl –(C=O)- (H_2_BPHD), or sulfonyl -S(O)_2_- (H_2_SDBA) functional groups. These efforts have afforded a series of Cu^2+^ [99], Zr^4+^ [20] and trivalent rare earth MOFs [34,72] with interesting structural, sorption, and sensing properties. These investigations have been recently extended to tetracarboxylic V-shaped ligands, and, in this work, we report three new compounds synthesized from the use of the diphthalic ligand H_4_STBA (Scheme S1a in Supplementary Materials). According to a survey of the literature and the CCDC database, MOFs comprising the deprotonated H_4−n_STBA^n−^ ligand without auxiliary bridging ligands containing N-donor atoms are relatively rare (a CCDC search returned fewer than 40 structures). This tetracarboxylic ligand has also been utilized in Cd^2+^-chemistry, yielding only a handful of MOF examples [36,54,64]. As such, we explored the chemistry of the H_4_STBA ligand in Cd^2+^- and AE^2+^-chemistry. These synthetic efforts and, in particular, the reaction of CdCl_2_·H_2_O with 3,3′,4,4′-DPSDA in DMF/H_2_O (5 mL/2 mL) afforded compound [NH_2_(CH_3_)_2_]^+^_2_[Cd(STBA)]^2−^_n_·nDMF·1.5nH_2_O—(**1**)_n_·nDMF·1.5nH_2_O, which represents an anionic framework where the negative charge is counterbalanced by two [NH_2_(CH_3_)_2_]^+^ cations. Considering this result, it was decided to use a second, divalent metal ion in the reaction mixture with the aim of isolating neutral heterometallic MOFs. The exact same reaction that led to (**1**)_n_·nDMF·1.5nH_2_O was repeated with the difference that an AE(NO_3_)_2_ salt was employed in the reaction mixture. These efforts led to compounds [CdCa(STBA)(H_2_O)_2_]_n_·0.5nDMF—(**2**)_n_·0.5nDMF and [CdSr(STBA)(H_2_O)_2_]_n_·0.5nDMF—(**3**)_n_·0.5nDMF, which are neutral MOFs.

### 3.2. Structural Characterization

**Figure 1:**
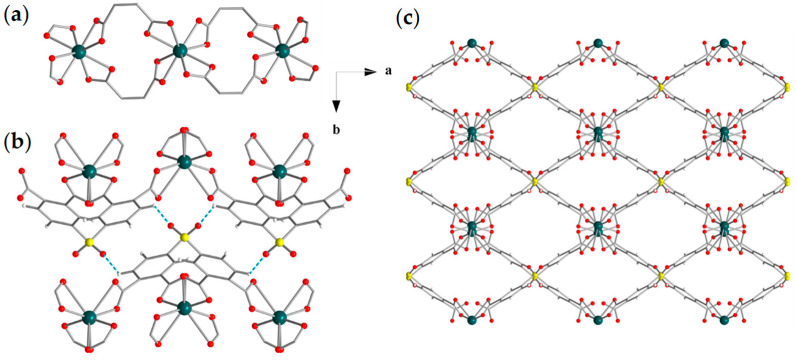
Representations of (**a**) part of the 1D chain SBU, (**b**) the inter-layer connection of the 2D nanosheets through hydrogen bonds involving the hydrogen atom of a benzene ring and the O atoms of the sulfonyl functional group of the STBA^4−^ ligand along a axis and (**c**) the 2D nanosheets along the c axis showing the rhombic channels defined by two STBA^4−^ anions and two chain SBUs of (**1**)_n_. Color code: Cd, green; S, yellow; O, red; C, gray; H, white.

Representations of the crystal structure of compound (**1**)_n_, crystallizing in the monoclinic space group *P2/c*, are illustrated in Figure 1, and selected crystal data and bond lengths are listed in Tables S1 and S2, respectively, in the Supplementary Materials. The asymmetric unit comprises a half cadmium ion and a half STBA^4−^ ligand. The coordination environment of Cd1 ion comprises eight O atoms of four carboxylic groups, chelating to the metal ion, of four different STBA^4−^ ligands, adopting a Johnson Gyrobifastigium (J26) geometry [100]. There is one crystallographically independent STBA^4−^ ligand that connects four metal ions in a η^1^:η^1^:η^1^:η^1^:η^1^:η^1^:η^1^:η^1^:μ_4_ fashion (Scheme S1b in the Supplementary Materials). The connection of the Cd^2+^ ions through the COO^−^ groups of STBA^4−^ ligands leads to a 1D chain along the crystallographic c axis with the formula [Cd(COO^−^)_4_]^2−^, which is the secondary building unit (SBU) of (**1**)_n_ (Figure 1a). The chains are linked through the STBA^4−^ anions, resulting in a 2D network (Figure 1b). A thorough inspection of the packing of (**1**)_n_ showed the existence of rhombic channels along the c axis, defined by two STBA^4−^ anions and two chain SBUs (Figure 1c). Moreover, soft inter-layer hydrogen-bonding interactions are observed between the hydrogen atom of a benzene ring in one STBA^4−^ ligand (donor) and the O atom of the sulfonyl group, -S(O)_2_-, in another STBA^4−^ ligand (acceptor) (O···C distance ≈ 3.24 Å) which lead to the parallel packing of the 2D sheets and the formation of a pseudo 3D MOF structure (Figure 1b) [72,99].

The total charge of (**1**)_n_ is negative and is counterbalanced by two dimethylammonium cations formed from the in situ decomposition of DMF molecules under elevated temperature and pressure conditions. The solvent-accessible volume (SAV) for (**1**)_n_ was calculated, using program PLATON, to 50% of the unit cell volume.

**Figure 2:**
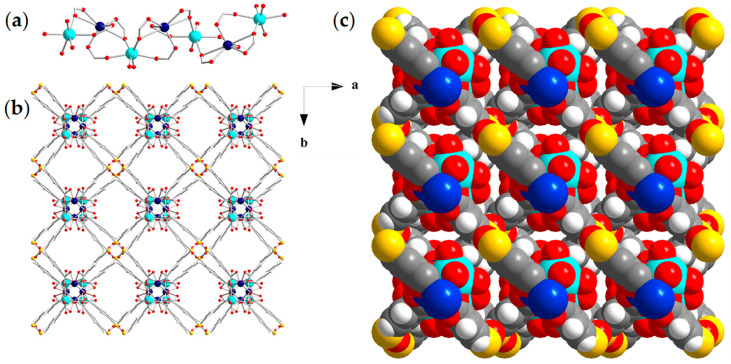
Representations of (**a**) the 1D [CdCa(COO^−^)_4_] helical chain SBU, (**b**) the connection of [CdCa(COO^−^)_4_] SBUs via STBA^4−^ ligands along c axis and (**c**) the space-filling model of compound (**2**)_n_ along c axis. Color code: Cd, dark blue; Ca, turquoise; S, yellow; O, red; C, gray; H, white.

Representations of the crystal structure of compound (**2**)_n_, crystallizing in the tetragonal space group *P4_1_22*, are illustrated in Figure 2, and selected crystal data and bond lengths are listed in Tables S1 and S3, respectively, in the Supplementary Materials. The asymmetric unit comprises a half cadmium ion, a half calcium ion, and a half STBA^4−^ ligand. Its SBU contains a 1D helical chain with molecular formula [CdCa(COO^−^)_4_] along the crystallographic c-axis (Figure 2a). The coordination environment of the crystallographically independent Cd^2+^ center contains six carboxylate O atoms of four different STBA^4−^ ligands, two of which bind to the metal ions in a chelating and the remaining two in a monodentate mode, adopting a distorted octahedral geometry [100], whereas the one of Ca^2+^ ion consists of four carboxylate O atoms of four different STBA^4−^ anions and two O atoms of terminal water molecules, adopting a distorted octahedral geometry [100]. There is one crystallographically independent STBA^4−^ ligand that connects eight metal centers in a η^2^:η^1^:η^1^:η^1^:η^2^:η^1^:η^1^:η^1^:μ_8_ fashion (Scheme S1c in the Supplementary Materials). The 1D SBUs are linked to four adjacent ones through STBA^4−^ anions forming, in this way, a 3D network (Figure 2b). A thorough inspection of the packing of (**2**)_n_ showed the existence of rhombic channels along the c axis (Figure 2b,c). The SAV for (**2**)_n_ was calculated to ~45% of the unit cell volume.

**Figure 3:**
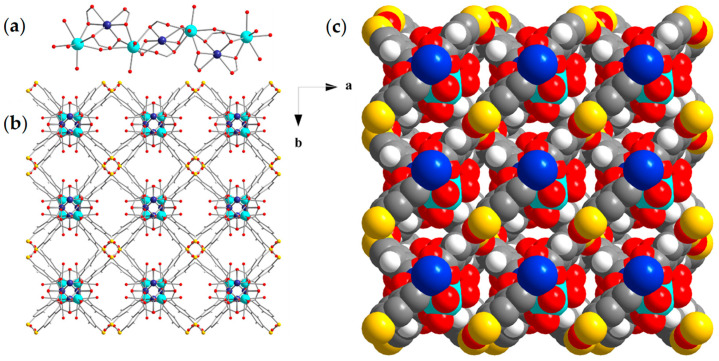
Representations of (**a**) the 1D [CdSr(COO^−^)_4_] helical chain SBU, (**b**) the connection of [CdSr(COO^−^)_4_] SBUs through STBA^4−^ ligands along c axis and (**c**) the space-filling model of compound (**3**)_n_ along c axis. Color code: Cd, dark blue; Sr, turquoise; S, yellow; O, red; C, gray; H, white.

Representations of the crystal structure of compound (**3**)_n_, crystallizing in the tetragonal space group *P4_3_22*, are illustrated in Figure 3, and selected crystal data and bond lengths are listed in Tables S1 and S4, respectively, in the Supplementary Materials. The asymmetric unit comprises a half cadmium ion, a half strontium ion, and a half STBA^4−^ ligand. Its SBU consists of a 1D helical chain with molecular formula [CdSr(COO^−^)_4_] along the crystallographic c-axis (Figure 3a). The coordination environment of the crystallographically independent Cd^2+^ ion consists of six carboxylate O atoms of four STBA^4−^ ligands, two of which bind to the metal ions in a chelating and the remaining two in a monodentate mode, adopting a distorted octahedral geometry [100]. The coordination environment of the Sr^2+^ center comprises six carboxylate O atoms of four STBA^4−^ ligands and two O atoms of water solvent molecules, adopting a triangular dodecahedral geometry [100]. There is one crystallographically independent STBA^4−^ ligand that connects eight metal ions in a η^1^:η^2^:η^2^:η^1^:η^1^:η^2^:η^2^:η^1^:μ_8_ fashion (Scheme S1d in the Supplementary Materials). The one-dimensional SBUs are linked to four adjacent ones through STBA^4−^ ligands, resulting in a three-dimensional network (Figure 3b). A thorough inspection of the packing of (**3**)_n_ showed the existence of rhombic channels along the c-axis (Figure 3b,c). The SAV of (**3**)_n_ corresponds to ~44% of the unit cell volume.

### 3.3. Physical Characterization

The stability of the reported MOFs in selected solvents was examined by pXRD. These studies revealed that the MOFs retain their structural integrity and crystallinity upon exposure to air as well as after treatment with most organic solvents, despite their relatively high solvent-accessible volumes (Figures S1–S3). Also, their IR spectra are shown in Figures S4–S6, and the assignment of selected IR bands is summarized in Tables S5–S7.

The thermal stability of microcrystalline samples of the reported MOFs, treated with acetonitrile, was studied with thermogravimetric analysis (Figures S7–S9) and VT-pXRD (Figures S10–S12). Their thermal decomposition includes continuous mass losses. These are attributed to the removal of terminally ligated and guest solvent molecules (H_2_O/DMF) that is completed at temperatures up to ~290 °C and the combustion of the tetracarboxylic ligand that is completed at ~560–570 °C. The residual mass at 900 °C corresponds to CdO for compound (**1**)_n_ and an equimolar mixture of CdO/CaO for compound (**2**)_n_ and CdO/SrO for compound (**3**)_n_ (Figures S7–S9). A more detailed discussion of the TGA studies for each MOF is included in Supplementary Materials. VT-pXRD studies revealed that compounds (**1**)_n_–(**3**)_n_ retain their crystallinity and structural integrity up to ~350–400 °C, depending on the compound (Figures S10–S12). The morphological features of the compounds were investigated by field-emission scanning electron microscopy (FE-SEM). The results indicate rod-shaped crystals for compound (**1**)_n_ with an average particle size of 17 μm (Figure S13). Compound (**2**)_n_ showed larger rod-like aggregates with particle sizes ranging from 40 to 492 μm (Figure S14), whereas compound (**3**)_n_ displayed needle-like particles with partial aggregation and an average particle size of 46 μm (Figure S15). Energy-dispersive X-ray spectroscopy (EDS) analytical results are in fair agreement with the elemental composition determined from single-crystal X-ray crystallography, yielding a Cd/Ca atomic ratio of ~1.2 for compound (**2**)_n_, and a Cd/Sr atomic ratio of ~0.8 for compound (**3**)_n_ (Figures S14C and S15D).

### 3.4. Gas Adsorption Measurements

**Figure 4:**
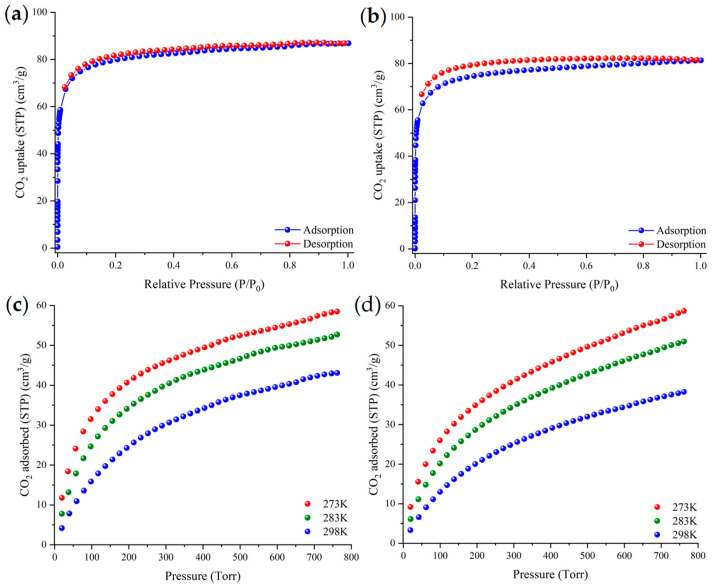
CO_2_ adsorption isotherms measured at 195 K for (**a**) (**2**)_n_ and (**b**) (**3**)_n_, and CO_2_ adsorption isotherms measured at 273 K, 283 K, and 298 K for (**c**) (**2**)_n_ and (**d**) (**3**)_n_.

The large SAVs observed in compounds (**2**)_n_ and (**3**)_n_ led us to explore their gas adsorption properties. Activation of the two compounds was achieved by replacing both lattice and coordinated solvent molecules with acetonitrile (see experimental part/gas adsorption in the Supplementary Materials). The activated samples of (**2**)_n_ and (**3**)_n_ retain their crystallinity and structural integrity as confirmed by pXRD studies (Figures S16 and S17). Carbon dioxide adsorption measurements for activated materials (**2**)_n_ and (**3**)_n_ at 195 K exhibited type-I isotherms (Figure 4a,b), typical for microporous materials, from which the apparent BET surface areas were determined to 370 m^2^/g (Langmuir area, 403 m^2^/g) and 343 m^2^/g (Langmuir area, 375 m^2^/g) (Figures S18–S21), respectively. The total pore volume values calculated at relative pressure, p/p_0_ = 0.995, are 0.109 cm^3^/g and 0.102 cm^3^/g for compounds (**2**)_n_ and (**3**)_n_, respectively. These pore volume values are smaller than those calculated by PoreBlazer [101], most likely due to the partial blockage of the pores by residual organic solvent molecules.

The CO_2_ uptake of these MOFs at 1 bar was found to be 2.58, 2.32, and 1.90 mmol/g for compound (**2**)_n_ and 2.58, 2.24, and 1.68 mmol/g for compound (**3**)_n_ at 273 K, 283 K, and 298 K, respectively (Figure 4c,d). These values are comparable with those reported for other MOFs based on related ligands/or metal ions [8,9]. The isosteric heat of adsorption, Qst, was determined for (**2**)_n_ and (**3**)_n_ to 28.4 and 26.2 kJ/mol at zero coverage (Qst^0^), respectively (Figures S22–S25). These values fall within the expected range for microporous Cd^2+^-MOFs [102].

## 4. Conclusions

Summarizing, a homometallic Cd^2+^-MOF and heterometallic Cd^2+^/AE^2+^-MOFs (AE^2+^ = Ca^2+^ and Sr^2+^) are reported. Compound (**1**)_n_·nDMF·1.5nH_2_O is a charged, 2D anionic MOF, counter-balanced by two [NH_2_(CH_3_)_2_]^+^ cations, based on a homometallic helical Cd^2+^-chain SBU, whereas compounds (**2**)_n_·0.5nDMF and (**3**)_n_·0.5nDMF are neutral 3D MOFs, based on heterometallic helical Cd^2+^/AE^2+^-chain SBUs. The two heterometallic compounds were targeted after the synthesis and structural characterization of (**1**)_n_·nDMF·1.5nH_2_O was completed due to the existence of two positively charged cations in the structure of (**1**)_n_·nDMF·1.5nH_2_O and polytopic ligands favoring the formation of heterometallic MOFs. Interestingly, compounds (**2**)_n_·0.5nDMF and (**3**)_n_·0.5nDMF were isolated from the same reaction that afforded (**1**)_n_, with the only modification being the inclusion of AE(NO_3_)_2_ (AE^2+^ = Ca^2+^ (**2**)_n_, or Sr^2+^ (**3**)_n_) in the reaction mixture. The structural differences between these three compounds afforded from analogous synthetic procedures highlight the structure-directing capability mainly of the alkaline earth heterometal ions, which is attributed to their flexible coordination sphere. Notably, these compounds are the initial heterometallic M^n+^/AE^2+^ (M = any metal ion) MOFs containing ligand H_4_STBA or its deprotonated analogs. In addition, all three compounds exhibit appreciable thermal and chemical stability, retaining their crystallinity and structure after exposure to a range of organic solvents and at elevated temperatures (up to 350–400 °C) as evidenced by thermogravimetric analysis and variable temperature powder X-ray diffraction measurements. Gas adsorption measurements on activated materials (**2**)_n_ and (**3**)_n_ showed moderate BET areas of 370 m^2^/g (Langmuir, 403 m^2^/g) and 343 m^2^/g (Langmuir, 375 m^2^/g), respectively, and the capability to adsorb CO_2_ (2.58 mmol/g), at 273 K/1 bar for both compounds. Overall, this work highlights the capability of diphthalic tetracarboxylic ligands to stabilize homometallic Cd^2+^ and heterometallic M^n+^/AE^2+^ (M = any metal ion) microporous MOFs. Ongoing work is focused on developing functional materials from this versatile family of ligands.

## Data Availability

The original contributions presented in this study are included in the article/Supplementary Materials. Further inquiries can be directed to the corresponding author.

## Supplementary Materials

The following supporting information can be downloaded at: https://www.mdpi.com/article/10.3390/ma18204647/s1, Table S1: selected crystal data for (**1**)_n_, (**2**)_n_ and (**3**)_n_; Table S2: selected bond lengths of compound (**1**)_n_; Table S3: selected bond lengths of compound (**2**)_n_; Table S4: selected bond lengths of compound (**3**)_n_; Table S5: selected absorption bands of 3,3′,4,4′-DPSDA and compound (**1**)_n_; Table S6: selected absorption bands of 3,3′,4,4′-DPSDA and compound (**2**)_n_; Table S7: selected absorption bands of 3,3′,4,4′-DPSDA and compound (**3**)_n_; Scheme S1: (a) the angular dianhydride that was employed in the reaction mixtures afforded compounds (**1**)_n_, (**2**)_n_ and (**3**)_n_ (the tetracarboxylic ligand was formed from the in situ hydrolysis of the dianhydride) and the coordination modes of STBA^4−^ ligand in compounds (b) (**1**)_n_, (c) (**2**)_n_ and (d) (**3**)_n_; Figure S1: powder X-ray diffraction patterns of the as-synthesized compound (**1**)_n_ treated in various organic solvents, along with the simulated from single-crystal X-ray data; Figure S2: powder X-ray diffraction patterns of the as-synthesized compound (**2**)_n_ treated in various organic solvents, along with the simulated from single-crystal X-ray data; Figure S3: powder X-ray diffraction patterns of the as synthesized compound (**3**)_n_ treated in various organic solvents and H_2_O, along with the simulated from single-crystal X-ray data; Figure S4: IR spectra of 3,3′,4,4′-DPSDA and the as-synthesized compound (**1**)_n_; Figure S5: IR spectra of 3,3′,4,4′-DPSDA and the as-synthesized compound (**2**)_n_; Figure S6: IR spectra of 3,3′,4,4′-DPSDA and the as-synthesized compound (**3**)_n_; Figure S7: TGA graph of the as-synthesized compound (**1**)_n_; Figure S8: TGA graph of the as-synthesized compound (**2**)_n_; Figure S9: TGA graph of the as-synthesized compound (**3**)_n_; Figure S10: variable temperature powder X-ray diffraction patterns recorded under Ar flow of the compound (**1**)_n_ treated with acetonitrile; Figure S11: variable temperature powder X-ray diffraction patterns recorded under Ar flow of the compound (**2**)_n_ treated with acetonitrile; Figure S12: variable temperature powder X-ray diffraction patterns recorded under Ar flow of the compound (**3**)_n_ treated with acetonitrile; Figure S13: (a,b) FE-SEM images, (c) particle size distribution histogram with the Gaussian fitting (solid red line) and d) EDS spectrum of compound (1)_n_ treated with acetonitrile; Figure S14: (a,b) FE-SEM images and (c) EDS spectrum of as-synthesized compound (2)_n_ treated with acetonitrile; Figure S15: (a,b) FE-SEM images, (c) particle size distribution histogram with the Gaussian fitting (solid red line) and (d) EDS spectrum of compound (3)_n_ treated with acetonitrile; Figure S16: powder X-ray diffraction patterns of the as-synthesized, treated with acetonitrile and activated (collected after the completion of gas sorption studies) compound (**2**)_n_; Figure S17: powder X-ray diffraction patterns of the as-synthesized, treated with acetonitrile and activated (collected after the completion of gas sorption studies) compound (**3**)_n_; Figure S18: BET plot from CO_2_ adsorption isotherm at 195 K for compound (**2**)_n_; Figure S19: BET plot from CO_2_ adsorption isotherm at 195 K for compound (**3**)_n_; Figure S20: Langmuir plot from CO_2_ adsorption isotherm at 195 K for compound (**2**)_n_; Figure S21: Langmuir plot from CO_2_ adsorption isotherm at 195 K for compound (**3**)_n_; Figure S22: virial-type fitting of CO_2_ adsorption isotherms of compound (**2**)_n_ at 273 K, 283 K and 298 K according to Equation (S1); Figure S23: virial-type fitting of CO_2_ adsorption isotherms of compound (**3**)_n_ at 273 K, 283 K and 298 K according to Equation (S1); Figure S24: CO_2_ isosteric heat of adsorption in compound (**2**)_n_ as a function of surface coverage; Figure S25: CO_2_ isosteric heat of adsorption in compound (**3**)_n_ as a function of surface coverage. References [103,104,105,106,107,108,109] are cited in the Supplementary Materials.
