# Supplementary material for: Homometallic 2D Cd2+ and Heterometallic 3D Cd2+/Ca2+, Cd2+/Sr2+ Metal–Organic Frameworks Based on an Angular Tetracarboxylic Ligand
Source: Materials (Basel). 2025 Oct 10;18(20):4647. doi: 10.3390/ma18204647 (PMC12565934; doi:10.3390/ma18204647)
Supplement: Supplementary file 1 [file materials-18-04647-s001.zip › materials-3857359-supplementary.pdf]

Supplementary Material
(SM)

Homometallic 2D Cd2+ and Heterometallic 3D Cd2+/Ca2+, Cd2+/Sr2+ Metal–Organic Frameworks Based on an Angular Tetracarboxylic Ligand

Rafail P. Machattos[a], Nikos Panagiotou[a], Vasiliki I. Karagianni[b], Manolis J. Manos[b], Eleni E. Moushi*[c] Anastasios J. Tasiopoulos*[a]

[a]Department of Chemistry, University of Cyprus, 1678 Nicosia, Cyprus, e-mail: atasio@ucy.ac.cy

[b]Department of Chemistry, University of Ioannina, 45110 Ioannina, Greece, emanos@uoi.gr

[c]Department of Life Sciences, European University Cyprus, 2404, Nicosia, e.moushi@euc.ac.cy

Experimental

Reagent-grade chemicals were obtained from commercial sources (Aldrich, Merck, Alfa Aesar, TCI, BLD Pharm, etc.) and used without further purification. All synthetic procedures were carried out in air.

$[NH_2(CH_3)_2]^+{}_2[Cd(STBA)]^{2-}{}_n$–(**1**)$_n$: In a 20 ml glass vial containing DMF, 5 ml was added to the dianhydride 3,3',4,4'-DPSDA (0.1 g, 0.28 mmol), and the resulting solution was placed in an ultrasonic bath for 5 min. Then, deionized water (2 ml), HNO_3 65% (25 μL, 0.023 g, 0.36 mmol) and $CdCl_2{\cdot}H_2O$ (0.040 g, 0.22 mmol) were subsequently added. The reaction mixture was sonicated again for 5 min, sealed with a plastic cap, and left undisturbed in an oven at 100 °C for 24 h. The colorless crystalline solid of (**1**)$_n$ formed was filtered off, washed several times with DMF and dried in vacuo. The reaction yield was ~50% based on $CdCl_2{\cdot}H_2O$. Anal. Calcd (**1**)$_n$·nDMF·1.5nH_2O: C 39.75; H 4.64; N 6.05; Found: C 39.52; H 4.52; N 6.38.

$[CdCa(STBA)(H_2O)_2]_n$·0.5nDMF–(**2**)$_n$·0.5nDMF: In a 20 ml glass vial containing DMF, 5 ml was added to the dianhydride 3,3',4,4'-DPSDA (0.1g, 0.28mmol) and the resulting solution was placed in an ultrasonic bath for 5 min. Then, deionized water (2 ml), HNO_3 65% (25 μL, 0.023 g, 0.36 mmol) and solids $CdCl_2{\cdot}H_2O$ (0.040g, 0.22 mmol) and $Ca(NO_3)_2{\cdot}4H_2O$ (0.048 g, 0.20 mmol) were subsequently added. The reaction mixture was sonicated again for 5 min, sealed with a plastic cap and left undisturbed in an oven at 100 °C for 24 h. The colorless crystalline solid of (**2**)$_n$ formed was filtered off, washed several times with DMF and dried in vacuo. The reaction yield was ~78% based on $Ca(NO_3)_2{\cdot}4H_2O$. Anal. Calcd (**2**)$_n$·0.5nDMF: C 34.16; H 2.21; N 1.14; Found: C 34.43; H 2.52; N 1.35.

$[CdSr(STBA)(H_2O)_2]_n$·0.5nDMF–(**3**)$_n$·0.5nDMF: In a 20 ml glass vial containing DMF, 5 ml was added the dianhydride 3,3',4,4'-DPSDA (0.1 g, 0.28 mmol) and the resulting solution was placed in an ultrasonic bath for 5 min. Then, deionized water (2 ml), HNO_3 65% (25 μL, 0.023 g, 0.36 mmol) and solids $CdCl_2{\cdot}H_2O$ (0.040 g, 0.22 mmol) and $Sr(NO_3)_2$ (0.042 g, 0.20 mmol) were subsequently added. The reaction mixture was sonicated again for 5 min, sealed with a plastic cap and left undisturbed in an oven at 100 °C for 24h. The colorless crystalline solid of (**3**)$_n$ formed was filtered off, washed several times with DMF and dried in vacuo. The reaction yield was ~75%

based on $Sr(NO_3)_2$. Anal. Calcd $(\mathbf{3})_n\cdot 0.5nDMF$: C 31.71; H 2.05; N 1.06; Found: C 31.90; H 2.32; N 1.34.

Stability evaluation

The stability of the as-synthesized compounds $(\mathbf{1})_n\cdot nDMF\cdot 1.5nH_2O$, $(\mathbf{2})_n\cdot 0.5nDMF$ and $(\mathbf{3})_n\cdot 0.5nDMF$ in acetonitrile was evaluated as follows: 50 mg of the MOF was suspended in 5 ml acetonitrile, and the resulting mixture was stirred for ~7 days while the solvent was replenished every 8 h. The solid was recovered and characterized by powder X-ray diffraction (pXRD).

Physicochemical characterization methods

Elemental analyses (C, H, N) were performed by the in-house facilities of the University of Cyprus, Chemistry Department. IR spectra were recorded on ATR in the 4000-700 cm^{-1} range using a Shimadzu Prestige-21 spectrometer. Powder X-ray diffraction patterns were recorded on a Rigaku Miniflex 6G X-ray diffractometer (Cu Kα radiation, λ = 1.5418Å). Variable temperature pXRD measurements (VT-pXRD) were performed on a BTS 500 high temperature attachment of the Rigaku Miniflex 6G X-ray diffractometer under Ar flow with an increase rate of 5 ^{o}C/min in the range of 25-500°C. Thermal stability studies were performed with a Shimadzu TGA 50 thermogravimetric analyzer. Scanning Electron Microscopy (SEM) combined with Energy-Dispersive Spectroscopy (EDS) was carried out using a Phenom Pharos G2 Desktop FEG-SEM (Thermo Fisher Scientific) equipped with an integrated EDS detector. Prior to analysis, the specimens were sputter-coated with a 5 nm chromium layer using a Q150T ES Plus automatic sputter coater (Quorum Technologies Ltd.) to minimize charging effects. The particle size distribution was determined using ImageJ software. Before determining particle size, the software was calibrated using the scale bar provided in the SEM images to ensure accurate size quantification. Individual particle diameters were then measured from the SEM images, and the resulting dataset was used to construct a histogram of particle sizes. A Gaussian fit was applied to the histogram to obtain the average particle size and standard deviation, thereby providing a reliable representation of the particle size distribution.

Single-crystal X-ray diffraction

Single-crystal X-ray diffraction studies were conducted using a Rigaku Supernova A diffractometer, equipped with a CCD area detector and employing Cu-Kα (λ = 1.5406 Å) and Mo-Ka (λ = 0.7107 Å) radiation, and a Rigaku XtaLAB Synergy S diffractometer, equipped with a HyPix-6000HE detector employing Cu-Kα (λ = 1.5406 Å) radiation. An appropriate single crystal was mounted on a cryoloop with paratone-N oil and transferred to a goniostat, where it was cooled for data collection. The structures were solved by direct methods using SHELXT and refined on F^2 using full-matrix least squares using SHELXL14.1 [103]. Software packages used: CrysAlis CCD for data collection [104], CrysAlis RED for cell refinement and data reduction, WINGX and Olex2 for geometric calculations [105,106] and DIAMOND for molecular graphics [107]. The disorder in the crystal structures was handled using various restraints (SIMU, RIGU, DELU, DFIX, ISOR). The non-H atoms were treated anisotropically, whereas the aromatic hydrogen atoms were placed in calculated, ideal positions and refined as riding on their respective carbon atoms. Electron density contributions from disordered guest molecules were handled using the SQUEEZE procedure from the PLATON software suite [ref]. Selected crystal data for $(\mathbf{1})_n$, $(\mathbf{2})_n$ and $(\mathbf{3})_n$ are summarized in Table S1, and bond lengths are summarized in Tables S2, S3 and S4, respectively, in the Supplementary Materials, SM. *Crystallographic data for* $(\mathbf{1})_n$, $(\mathbf{2})_n$ and $(\mathbf{3})_n$ have been deposited at the CCDC under accession numbers 2477739 - 2477741. Complete details are available in the CIF files provided as SM.

Table S1. Selected crystal data for (**1**)$_n$, (**2**)$_n$ and (**3**)$_n$.

Compound	(1)$_n$	(2)$_n$	(3)$_n$
Empirical Formula	$C_{16}H_6O_{10}SCd$	$C_{16}H_6O_{12}SCaCd$	$C_{16}H_6O_{12}SSrCd$
Formula Weight	502.67	574.75	622.29
Temperature	229.97(19) K	273(2) K	273(2) K
Wavelength	1.54184 Å	0.71073 Å	0.71073 Å
Crystal system	Monoclinic	Tetragonal	Tetragonal
Space group	P2/c	$P4_122$	$P4_322$
Unit cell dimensions	a = 12.8483(4)<br>b = 9.0354(2)<br>c = 12.5289(4)<br>α = γ =90°,<br>β=109.976(4)°	a = 11.4060(17)<br>b = 11.4060(17)<br>c = 23.298(3)<br>α = β = γ = 90°	a = 11.3197(10)<br>b = 11.3197(10)<br>c = 23.4502(16)<br>α = β = γ = 90°
Volume	1366.97(8) Å^3	3031.0(10) Å^3	3004.8(6) Å^3
Z	2	4	4
$d_{calc.}$/g/cm^3	1.221	1.259	1.376
Absorption Coefficient/ mm^{-1}	7.455	1.000	2.594
F (000)	492	1128	1200
Reflections collected	8635	16880	15640
Independent reflections	2659	3856	3779
Completeness to θ = 66.999°	99.8%<br>[R_{int} = 0.0294]	99.5%<br>[R_{int} = 0.1742]	99.6%<br>[R_{int} = 0.0774]
Data/ Restraints/ Parameters	2659 / 0 / 128	3856 / 81 / 132	3779 / 85 / 131
Goodness-of-fit	1.098	1.019	1.044
Final R indices [I> 2σ(I)]	$R_{obs.}$ = 0.0431, $wR_{obs.}$ = 0.1366	$R_{obs.}$ = 0.1165, $wR_{obs.}$ = 0.3115	$R_{obs.}$ = 0.1097, $wR_{obs.}$ = 0.3143
R indices [all data]	R_{all} = 0.0434, wR_{all} = 0.1370	R_{all} = 0.2576, wR_{all} = 0.3955	R_{all} = 0.1843, wR_{all} = 0.3672

$^aR = \Sigma||F_o|-|F_c|| / \Sigma|F_o|$, $wR = \{\Sigma[w(|F_o|^2 - |F_c|^2)^2] / \Sigma[w(|F_o|^4)]\}^{1/2}$ and

$^bw = 1/[\sigma^2(F_o^2)+(mP)^2+nP]$, where $P=(F_o^2+2F_c^2)/3$ and m and n are constants.

Table S2. Selected bond lengths of compound (**1**)$_n$.

Atoms 1	Atom 2	Distance (Å)
Cd1	O2'	2.574(4)
Cd1	O2''	2.574(4)
Cd1	O3'	2.312(3)
Cd1	O3''	2.312(3)
Cd1	O4	2.754(5)
Cd1	O4'''	2.754(5)
Cd1	O5	2.267(4)
Cd1	O5'''	2.267(4)

'; 1-x, 2-y, 1-z, ''; +x, 2-y, 1/2+z, '''; 1-x, +y, 3/2-z.

Table S3. Selected bond lengths of compound (**2**)$_n$.

Atoms 1	Atom 2	Distance (Å)
Cd1	O3	2.213(2)
Cd1	O3'	2.213(2)
Cd1	O4''	2.860(3)
Cd1	O4'''	2.860(3)
Cd1	O5''	2.285(2)
Cd1	O5'''	2.285(2)
Ca1	O2'	2.304(1)
Ca1	O2''''	2.304(1)
Ca1	O4	2.183(3)
Ca1	O4''	2.183(3
Ca1	O6	2.621(2)
Ca1	O6''	2.621(2)

'; 1-x, +y, 1-z, ''; 1-x, 1-y, 5/4-z, '''; 1-x, +y, -1/4+z, ''''; +x, 1-y, 1/4+z

Table S4. Selected bond lengths of compound (**3**)$_n$.

Atoms 1	Atom 2	Distance (Å)
Cd1	O3	2.286(2)
Cd1	O3'	2.286(2)
Cd1	O4''	2.480(2)
Cd1	O4'''	2.480(2)
Cd1	O5''	2.396(2)
Cd1	O5'''	2.396(2)
Sr1	O2	2.502(2)
Sr1	O2''''	2.502(2)
Sr1	O3'	2.876(2)
Sr1	O3''	2.876(2)
Sr1	O4'	2.542(3)
Sr1	O4''	2.542(3)
Sr1	O6	2.727(5)
Sr1	O6''''	2.727(5)

'; 1-x, +y, 1-z, ''; 1-x, +y, 1/4+z, '''; 1-x, 1-y, 3/4-z, ''''; +x, +y, 5/4-z

Gas adsorption

Low-pressure gas adsorption experiments were conducted at various temperatures using a Quantachrome Autosorb-iQ3 system equipped with a cryocooler, allowing simultaneous thermostatting of up to two samples in the 20 to 320K range. Prior to measurements, the as-synthesized materials were washed with *N,N*-dimethylformamide four times for one day, followed by soaking in acetonitrile. The acetonitrile was replaced three times daily for ten days. The resulting wet samples were placed into 6 mm sample cells and activated under dynamic vacuum at room temperature for 20 hours until the out-gassing rate dropped below 2 mTorr min^{-1}. After activation, the samples were reweighted to determine the exact mass of the activated materials, and the cells were transferred to the analysis manifold of the adsorption apparatus. High-purity Ar (99.999%), He (99.999%) and CO_2 (99.999%) gases were used in adsorption studies.

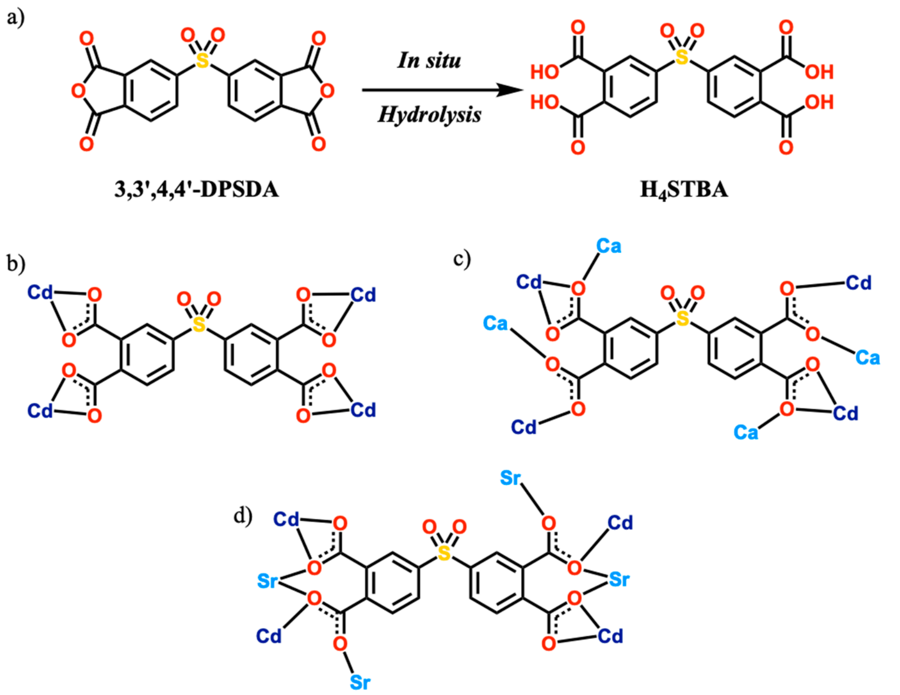

Scheme S1. a) The angular dianhydride that was employed in the reaction mixtures afforded compounds (**1**)$_n$, (**2**)$_n$ and (**3**)$_n$ (the tetracarboxylic ligand was formed from the in situ hydrolysis of the dianhydride) and the coordination modes of $STBA^{4-}$ ligand in compounds b) (**1**)$_n$, c) (**2**)$_n$ and d) (**3**)$_n$.

Physical Measurement/Characterization

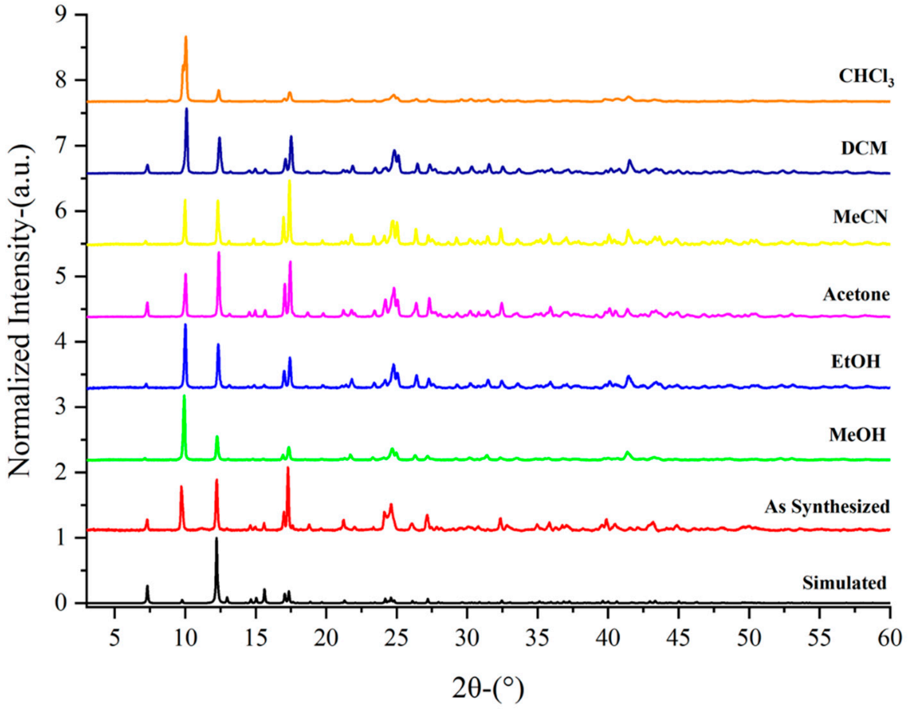

Figure S1. Powder X-ray diffraction patterns of the as-synthesized compound (**1**)$_n$ treated in various organic solvents, along with the simulated from single-crystal X-ray data.

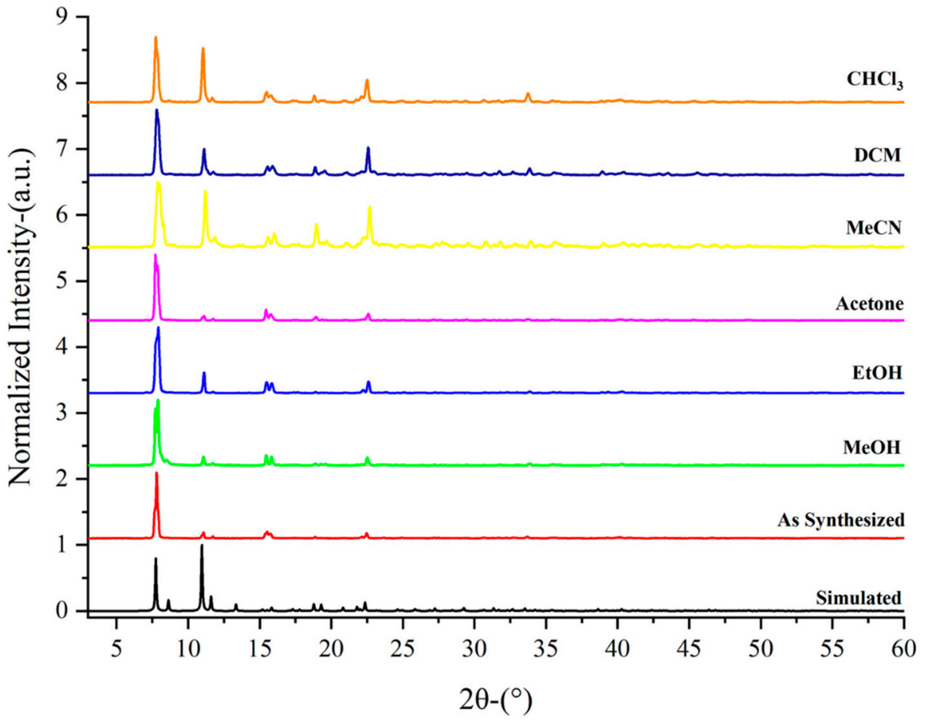

Figure S2. Powder X-ray diffraction patterns of the as-synthesized compound (**2**)$_n$ treated in various organic solvents, along with the simulated from single-crystal X-ray data.

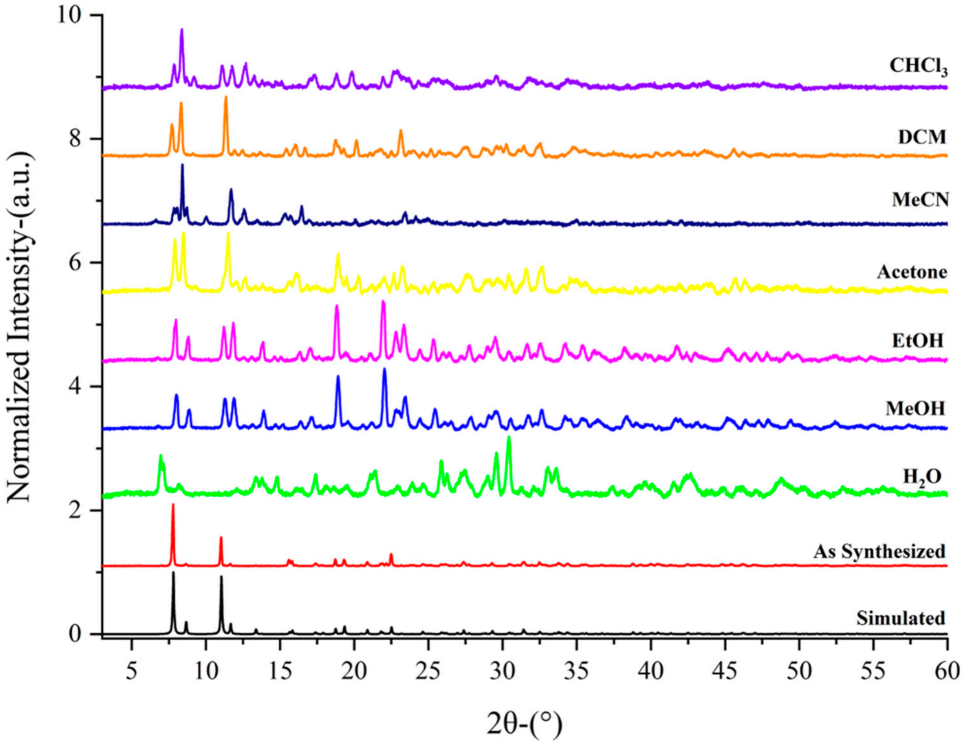

Figure S3. Powder X-ray diffraction patterns of the as-synthesized compound (**3**)$_n$ treated in various organic solvents and H_2O, along with the simulated from single-crystal X-ray data.

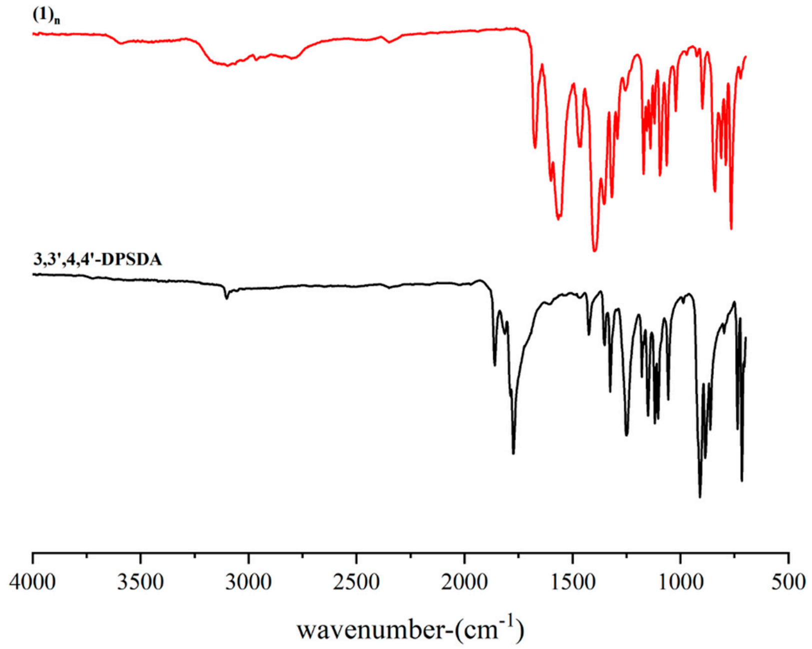

Figure S4. IR spectra of 3,3',4,4'-DPSDA and the as-synthesized compound (**1**)$_n$.

Table S5. Selected absorption bands of 3,3',4,4'-DPSDA and compound (**1**)$_n$.

**Vibration**	**Frequency (cm^{-1})**	
	**3,3',4,4'-DPSDA**	**(1)$_n$**
ν(=C-H, Ar.)	3006	2940
ν(C=O, Lig.)	1863	-
ν(C=O, Lig.)	1770	-
ν(C=O, DMF)	-	1674
ν(C=O, COO^-)$_{ant}$	-	1600
ν(C=O, COO^-)$_{sym}$	-	1352
ν(-SO_2-, Lig.)$_{ant}$	1325	1317
ν(-SO_2-, Lig.)$_{sym}$	1151	1130

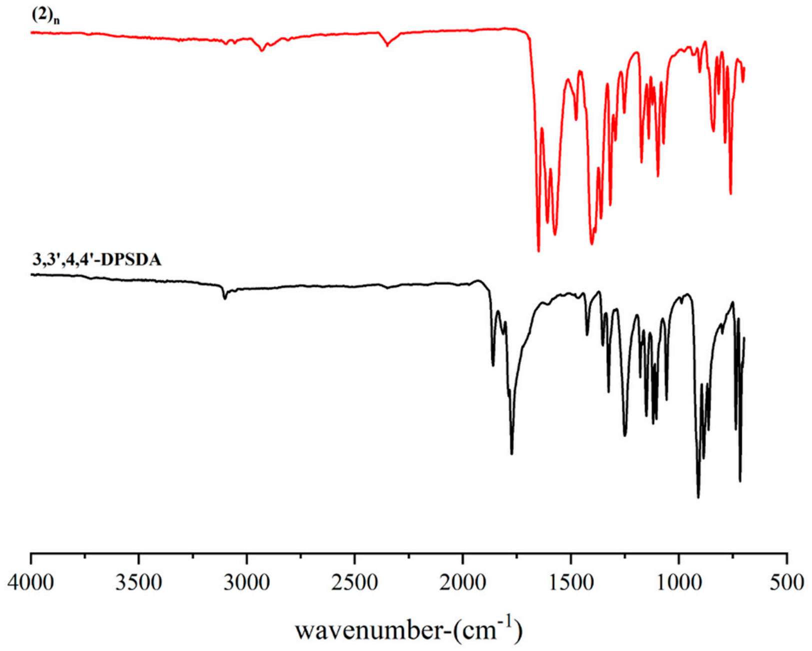

Figure S5. IR spectra of 3,3',4,4'-DPSDA and the as-synthesized compound (**2**)n.

Table S6. Selected absorption bands of 3,3',4,4'-DPSDA and compound (**2**)**n**.

Vibration	Frequency (cm^{-1})	
	**3,3',4,4'-DPSDA**	**(2)n**
ν(=C-H, Ar.)	3006	2931
ν(C=O, Lig.)	1863	-
ν(C=O, Lig.)	1770	-
ν(C=O, DMF)	-	1651
$\nu(C=O, COO^-)_{ant}$	-	1608
$\nu(C=O, COO^-)_{sym}$	-	1360
$\nu(-SO_2-, Lig.)_{ant}$	1325	1317
$\nu(-SO_2-, Lig.)_{sym}$	1151	1143

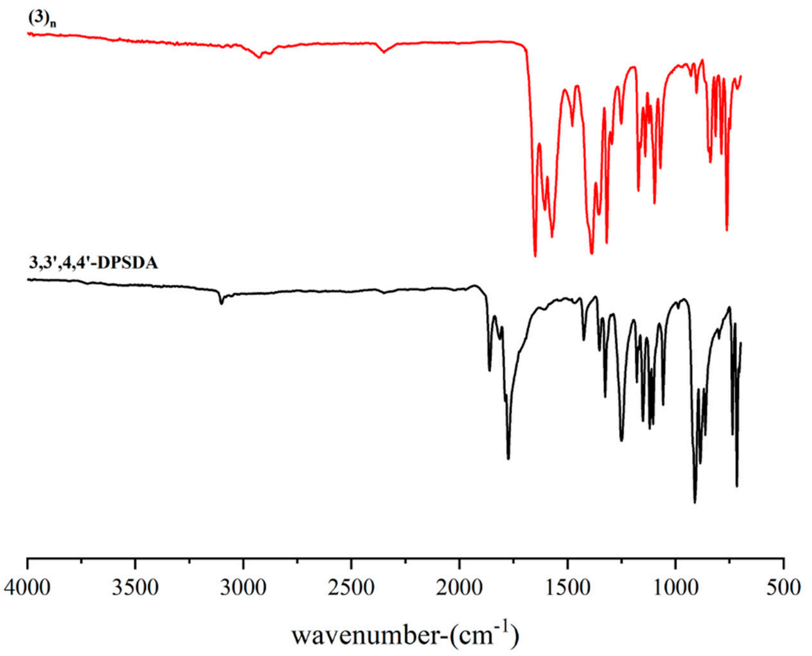

Figure S6. IR spectra of 3,3',4,4'-DPSDA and the as-synthesized compound (**3**)$_n$.

Table S7. Selected absorption bands of 3,3',4,4'-DPSDA and compound (**3**)$_n$.

Vibration	Frequency (cm^{-1})	
	**3,3',4,4'-DPSDA**	**(3)$_n$**
ν(=C-H, Ar.)	3006	2941
ν(C=O, Lig.)	1863	-
ν(C=O, Lig.)	1770	-
ν(C=O, DMF)	-	1651
ν(C=O, COO^-)$_{ant}$	-	1606
ν(C=O, COO^-)$_{sym}$	-	1355
ν(-SO_2-, Lig.)$_{ant}$	1325	1319
ν(-SO_2-, Lig.)$_{sym}$	1151	1145

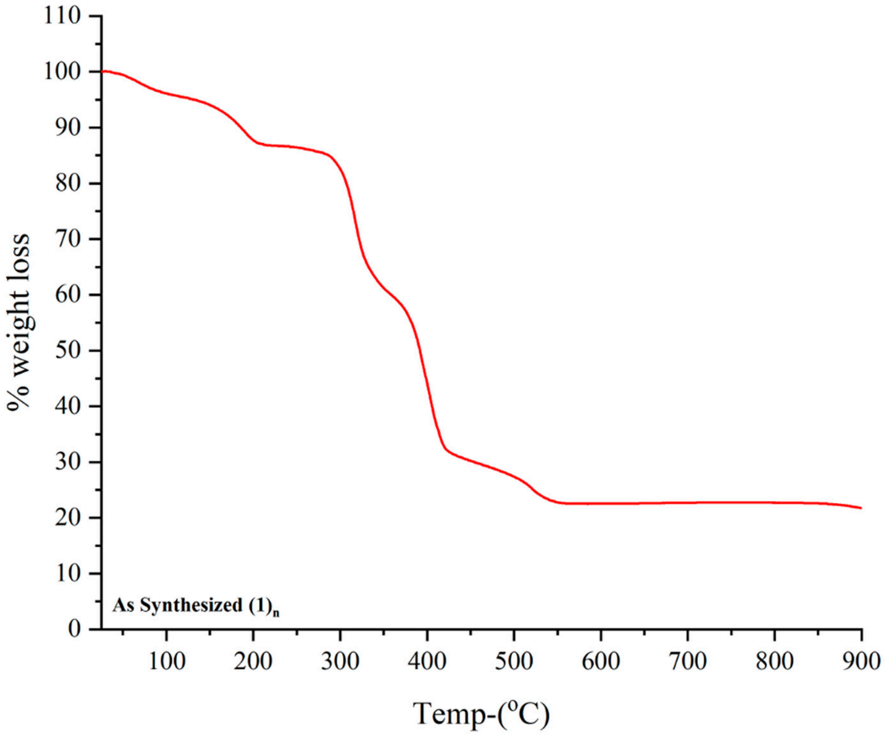

Figure S7. TGA graph of the as-synthesized compound (**1**)$_n$.

The TGA curve of (**1**)$_n$ revealed that the thermal decomposition of this compound proceeds via a multi-step process. A rough analysis of the TGA plot shall be provided below; however, there is uncertainty in this because the curve is quite complicated, containing several different mass losses (guest DMF and H_2O molecules and organic ligands). The release of guest solvent molecules (DMF + H_2O) involves continuous mass losses up to 290 °C, whereas the last mass loss at higher temperatures is due to the decomposition of the counter cation [$NH_2(CH_3)_2^+$] and the organic ligands $STBA^{4-}$. In particular, the mass losses in the temperature range of 25 °C to 290 °C correspond to ~13.5 % of the material's total mass and are attributed to the removal of one DMF and 1.5 H_2O guest molecules (calc. on the basis of the formula [$NH_2(CH_3)_2^+$]$_2$[Cd(STBA)]·nDMF·1.5nH_2O ((**1**)$_n$·nDMF·1.5nH_2O 14.4%)). The second mass loss, assigned to the decomposition of the organic ligand $STBA^{4-}$ and [$NH_2(CH_3)_2^+$], occurring in the ~ 290–570 °C region, corresponds to 65.0 % (calc. for (**1**)$_n$·nDMF·1.5nH_2O 67.1%) of the material's total mass. Lastly, the residual mass (21.5%) at 900 °C corresponds to CdO (calc. for (**1**)$_n$·nDMF·1.5nH_2O 18.5%).

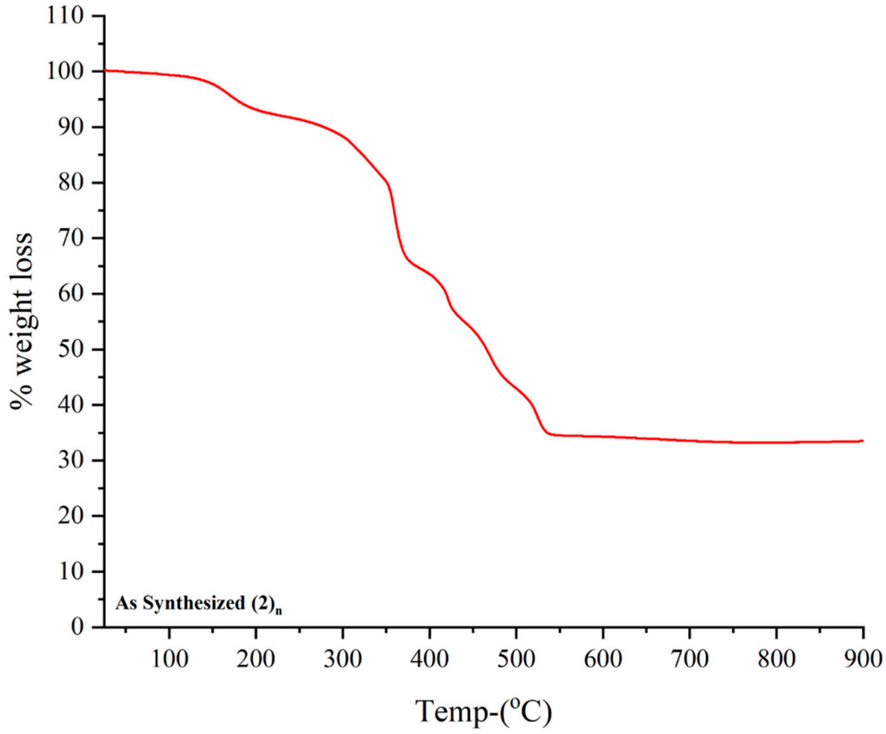

Figure S8. TGA graph of the as-synthesized compound (**2**)$_n$.

The TGA curve of (**2**)$_n$ revealed that the thermal decomposition of this compound proceeds via a multi-step process. A rough analysis of the TGA plot shall be provided below; however, there is uncertainty in this because the curve is quite complicated, containing several different mass losses (guest DMF and terminal H_2O molecules and organic ligand), some of which happen in the same temperature range, as evidenced by the lack of plateaus in the curve. The release of guest and coordinated solvent molecules (DMF + H_2O) involves continuous mass losses up to 290 °C, whereas the last mass loss at higher temperatures is due to the decomposition of the organic ligand $STBA^{4-}$. In particular, the mass losses in the temperature range 25 °C to 290 °C correspond to ~11.0 % of the material's total mass and are attributed to the removal of two terminally ligated H_2O and 0.5 guest DMF molecules (calc. on the basis of the formula $[CdCa(STBA)(H_2O)_2]_n$·0.5nDMF ((**2**)$_n$·0.5nDMF 11.8%)). The second mass loss, assigned to the decomposition of the organic ligand $STBA^{4-}$, occurring in the ~ 290 – 570 °C region, corresponds to 57.0 % (calc. for (**2**)$_n$·0.5nDMF 58.2%) of the material's total mass. Lastly, the residual mass (32.0 %) at 900 °C corresponds to CdO + CaO (calc. for (**2**)$_n$·0.5nDMF 30.0%).

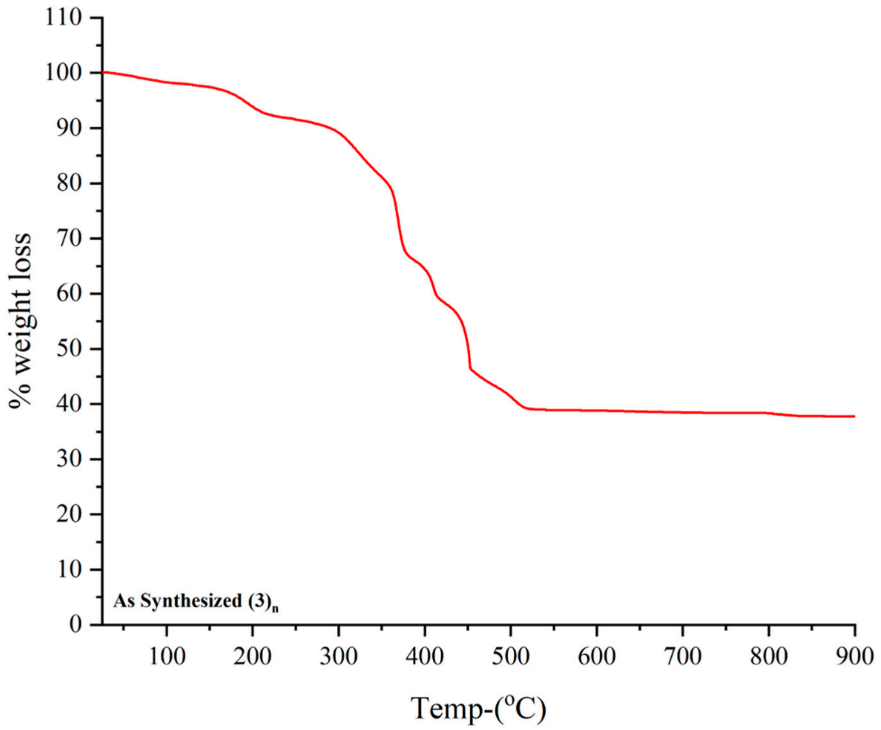

Figure S9. TGA graph of the as-synthesized compound $(\mathbf{3})_n$.

The TGA curve of $(\mathbf{3})_n$ revealed that the thermal decomposition of this compound proceeds via a multi-step process. A rough analysis of the TGA plot shall be provided below; however, there is uncertainty in this because the curve is quite complicated, containing several different mass losses (guest DMF and terminal H_2O molecules and organic ligand), some of which happen in the same temperature range, as evidenced by the lack of plateaus in the curve. The release of guest and coordinated solvent molecules (DMF + H_2O) involves continuous mass losses up to 290 °C, whereas the last mass loss at higher temperatures is due to the decomposition of the organic ligand $STBA^{4-}$. In particular, the mass losses in the temperature range of 25 °C to 290 °C correspond to ~10.0 % of the material's total mass and are attributed to the removal of two terminally ligated H_2O and 0.5 guest DMF molecules (calc. on the basis of the formula $[CdSr(STBA)(H_2O)_2]_n{\cdot}0.5nDMF$ ($(\mathbf{3})_n{\cdot}0.5nDMF$ 10.9%)). The second mass loss, assigned to the decomposition of the organic ligand $STBA^{4-}$, occurring in the ~ 290 – 560 °C region, corresponds to 53.0% (calc. for $(\mathbf{3})_n{\cdot}0.5nDMF$ 54.1 %) of the material's total mass. Lastly, the residual mass (37.0%) at 900 °C corresponds to CdO + SrO (calc. for $(\mathbf{3})_n{\cdot}0.5nDMF$ 35.0%).

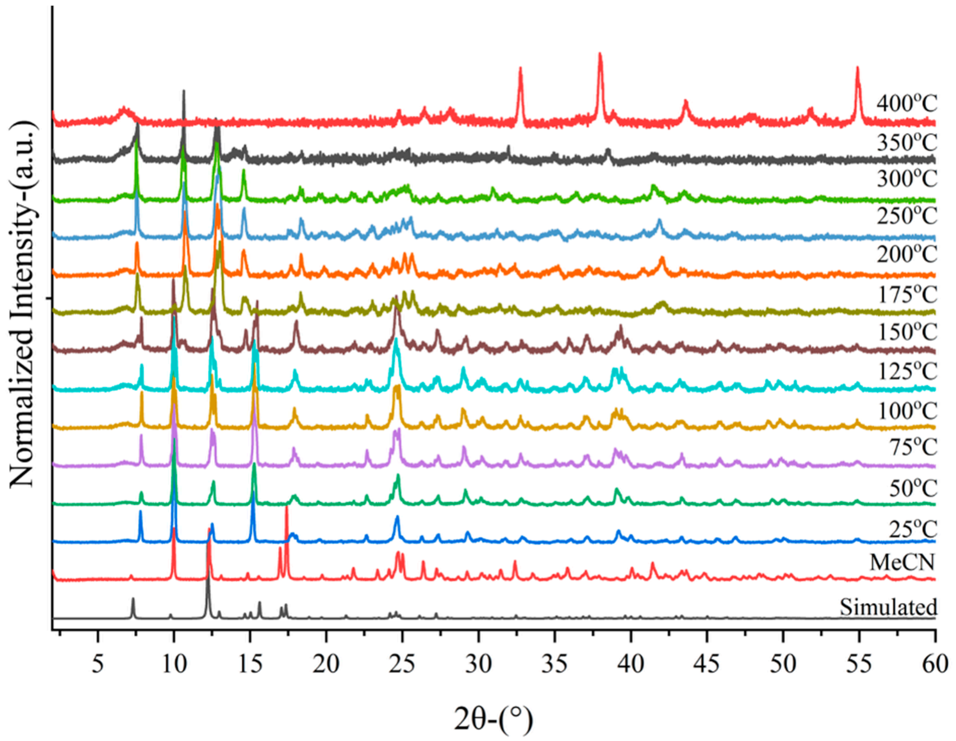

Figure S10. Variable temperature powder X-ray diffraction patterns recorded under Ar flow of the compound (**1**)$_n$ treated with acetonitrile.

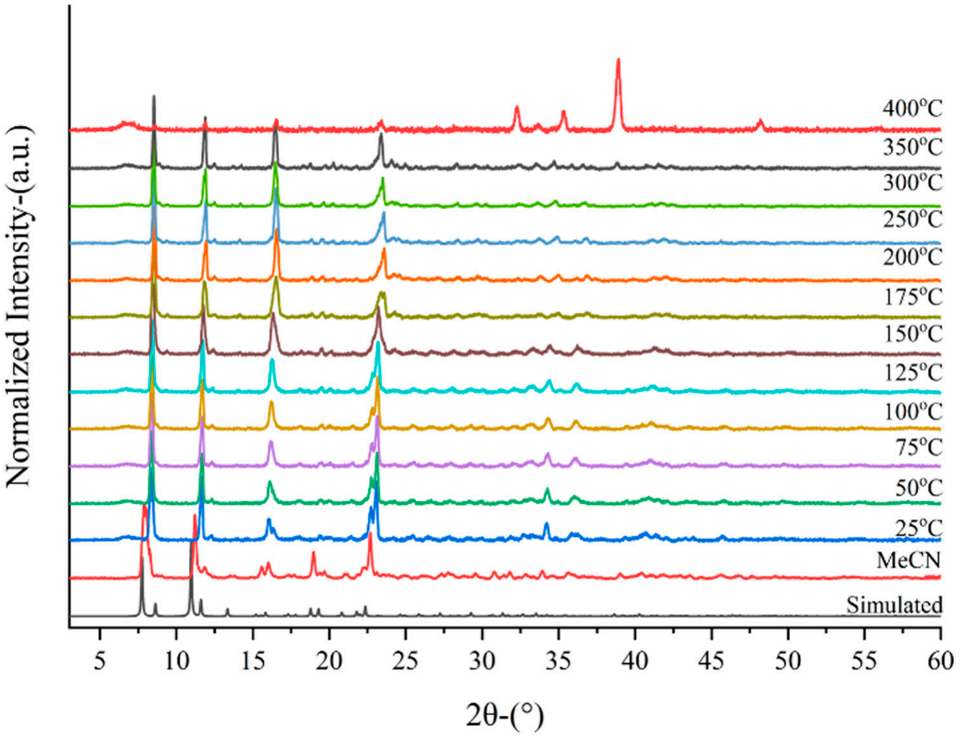

Figure S11. Variable temperature powder X-ray diffraction patterns recorded under Ar flow of the compound (**2**)$_n$ treated with acetonitrile.

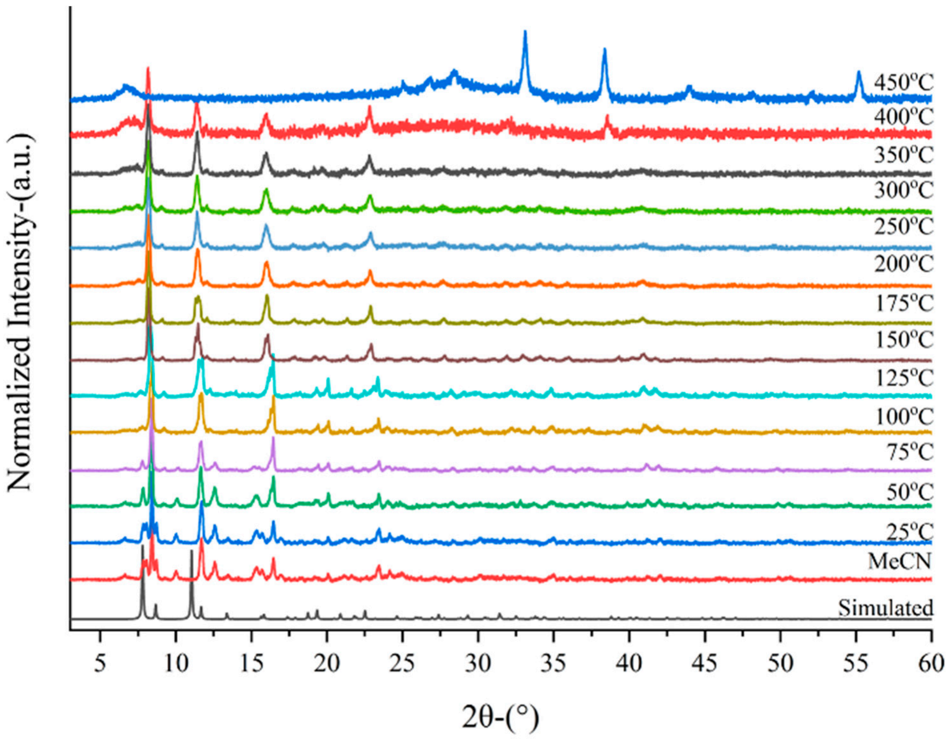

Figure S12. Variable temperature powder X-ray diffraction patterns recorded under Ar flow of the compound $(\mathbf{3})_n$ treated with acetonitrile.

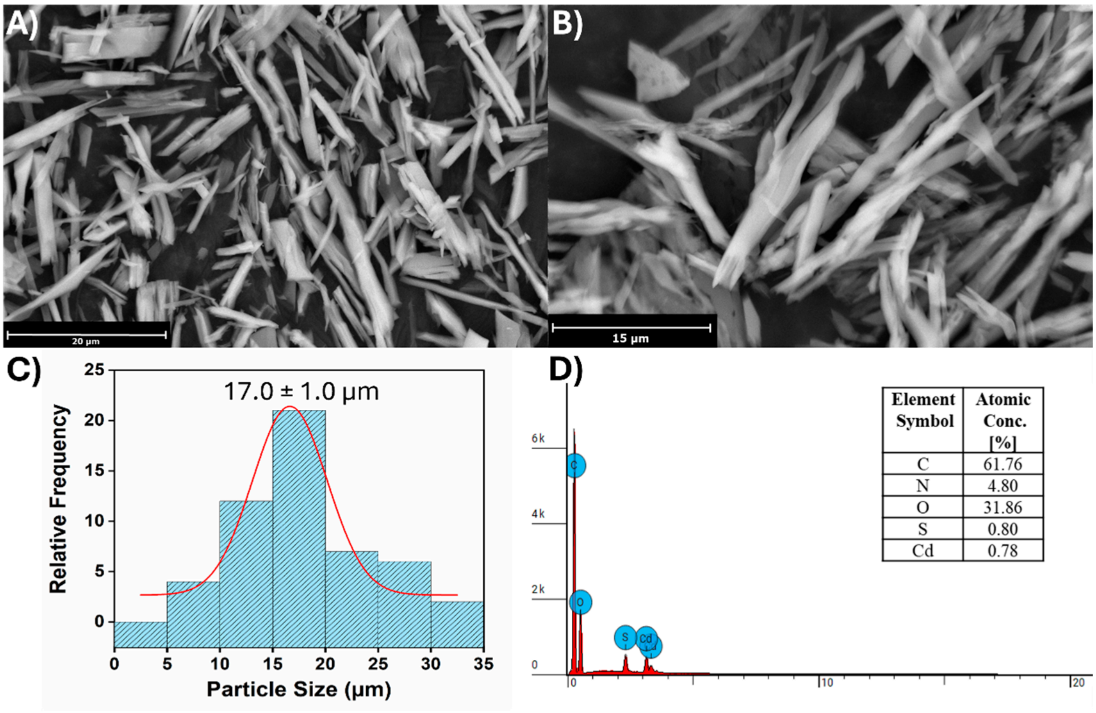

Figure S13. a,b) FE-SEM images, c) particle size distribution histogram with the Gaussian fitting (solid red line) and d) EDS spectrum of compound $(\mathbf{1})_n$ treated with acetonitrile.

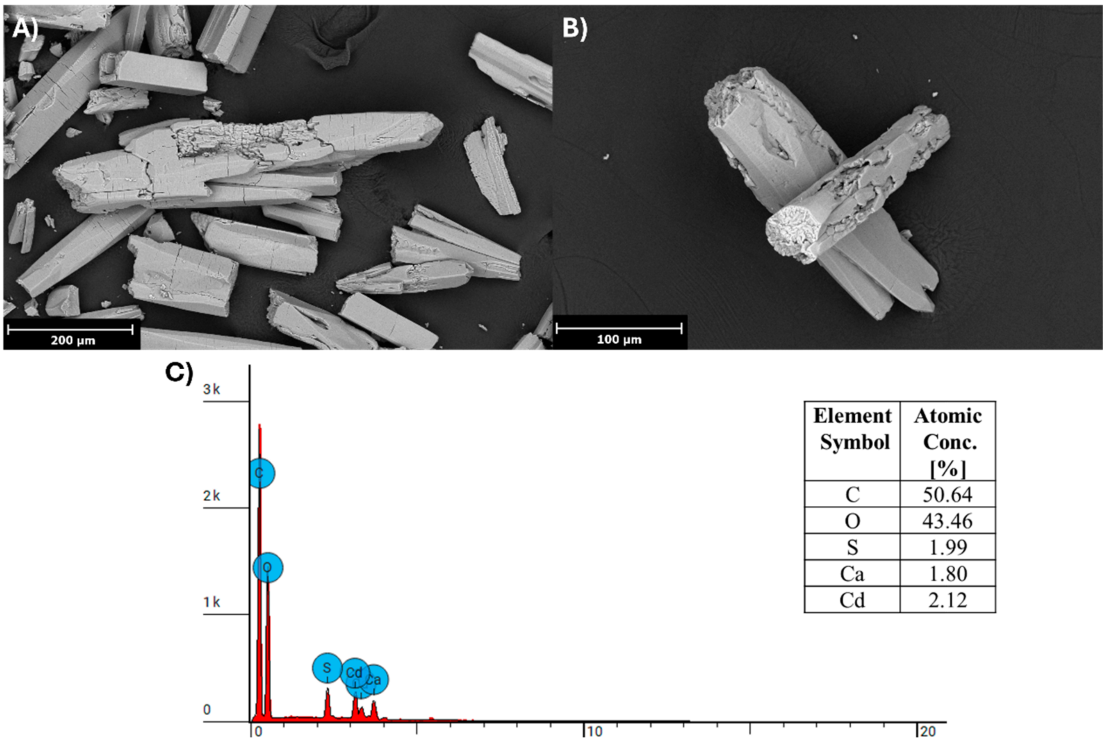

Element Symbol	Atomic Conc. [%]
C	50.64
O	43.46
S	1.99
Ca	1.80
Cd	2.12

Figure S14. a,b) FE-SEM images and c) EDS spectrum of as-synthesized compound ($\mathbf{2}$)$_n$ treated with acetonitrile.

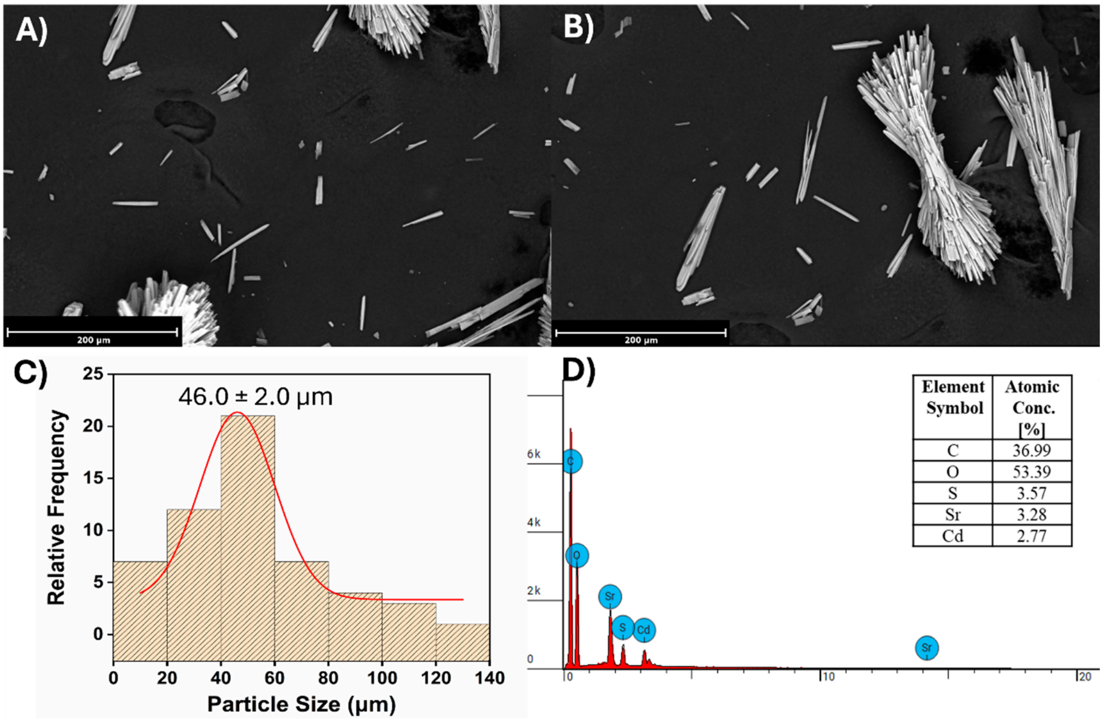

Element Symbol	Atomic Conc. [%]
C	36.99
O	53.39
S	3.57
Sr	3.28
Cd	2.77

Figure S15. a,b) FE-SEM images, c) particle size distribution histogram with the Gaussian fitting (solid red line) and d) EDS spectrum of compound ($\mathbf{3}$)$_n$ treated with acetonitrile.

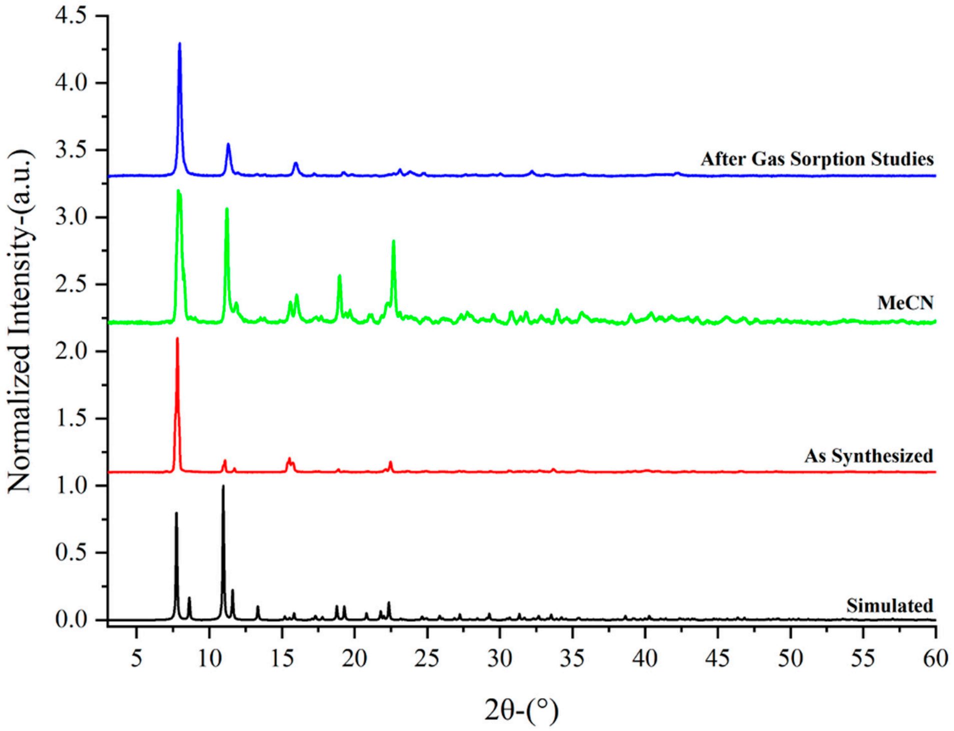

Figure S16. Powder X-ray diffraction patterns of the as-synthesized, treated with acetonitrile, and activated (collected after the completion of gas sorption studies) compound (**2**)$_n$.

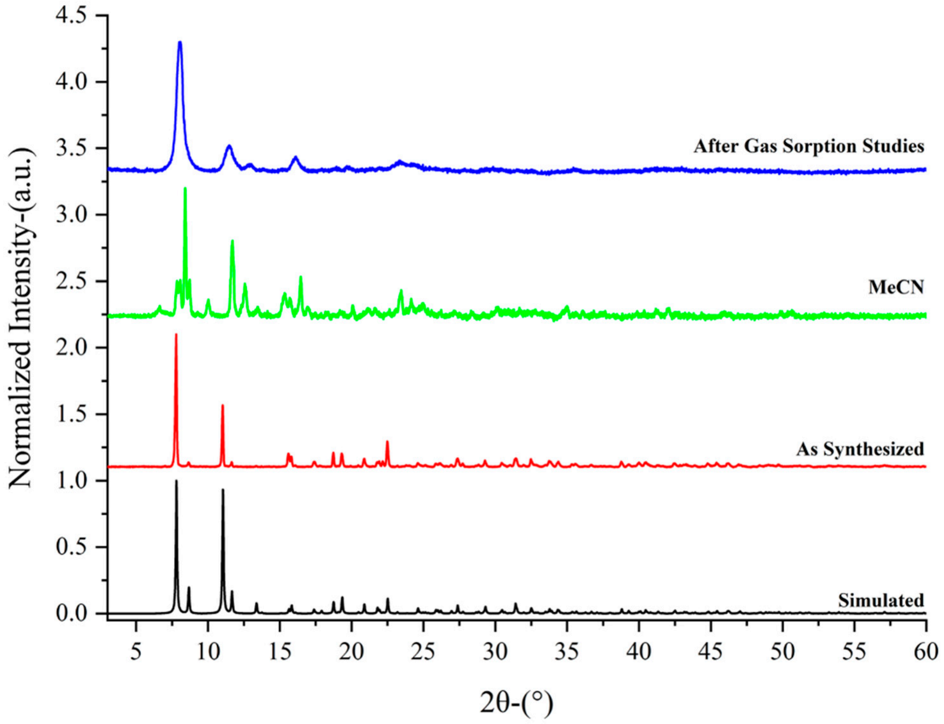

Figure S17. Powder X-ray diffraction patterns of the as-synthesized, treated with acetonitrile, and activated (collected after the completion of gas sorption studies) compound (**3**)$_n$.

Gas Sorption Measurements

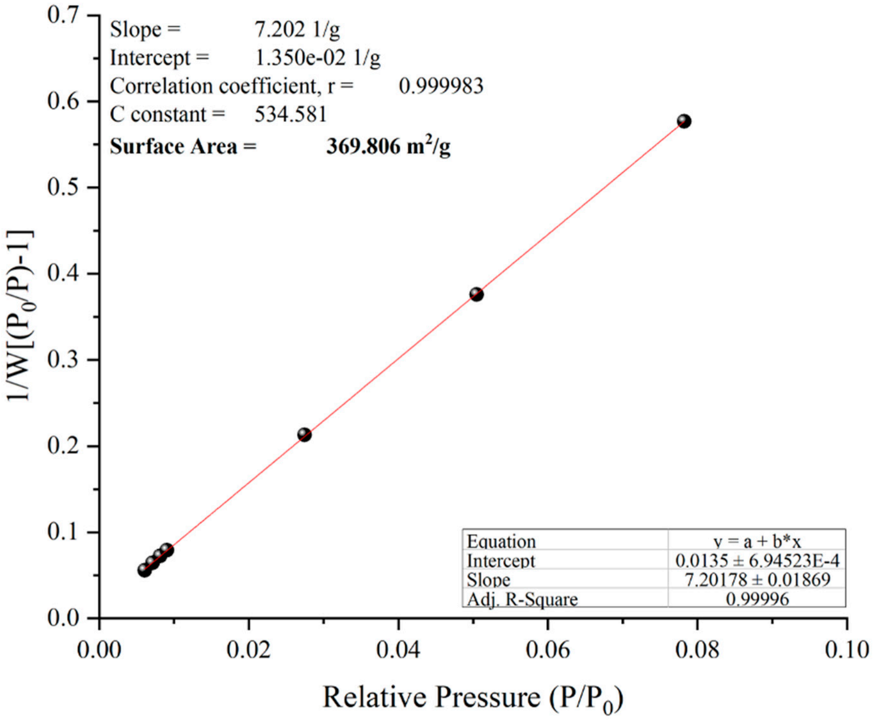

Figure S18. BET plot from CO_2 adsorption isotherm at 195K for compound (**2**)$_n$.

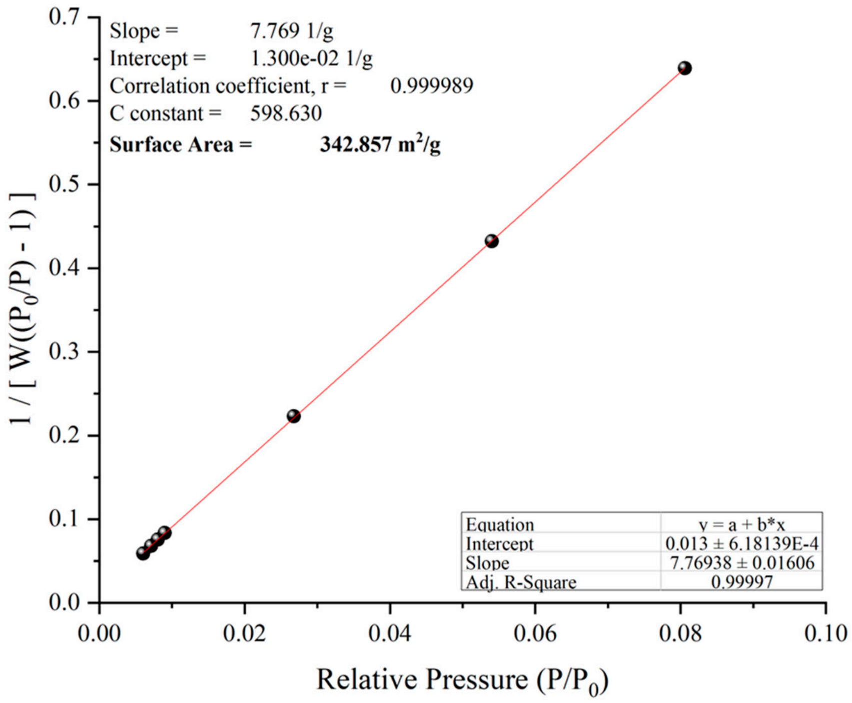

Figure S19. BET plot from CO_2 adsorption isotherm at 195K for compound (**3**)$_n$.

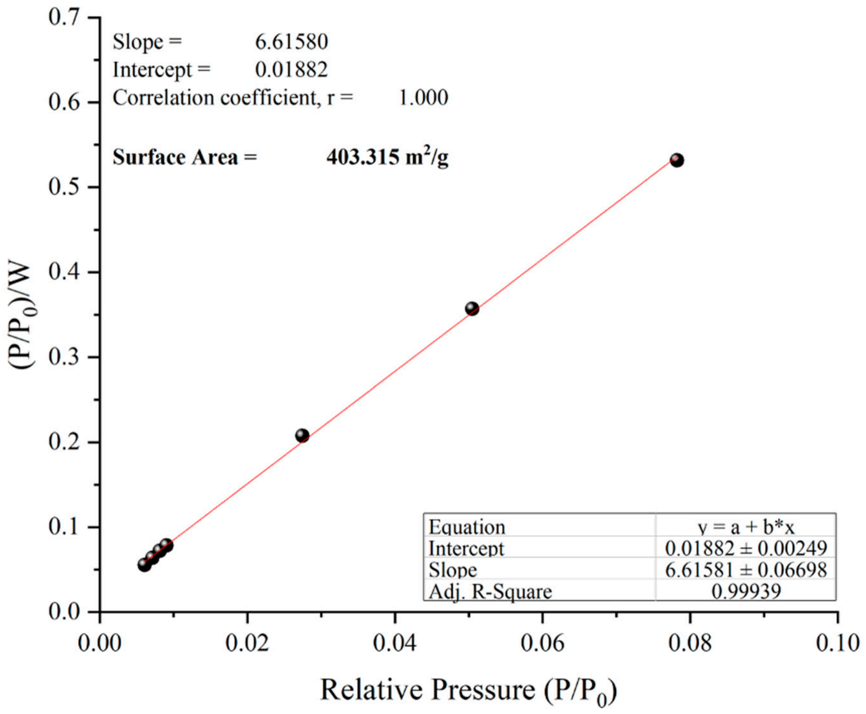

Figure S20. Langmuir plot from CO_2 adsorption isotherm at 195K for compound (**2**)$_n$.

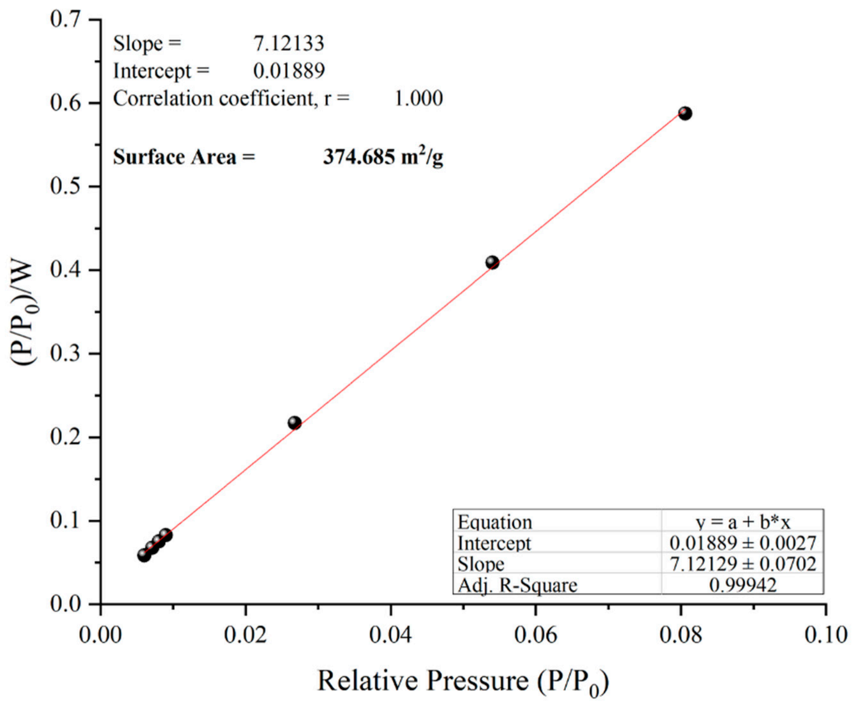

Figure S21. Langmuir plot from CO_2 adsorption isotherm at 195K for compound (**3**)$_n$.

Low-pressure CO_2 sorption isotherms and heat of adsorption calculations for CO_2.

Heat of adsorption: To calculate heat of adsorption, the corresponding CO_2 adsorption isotherms at three different temperatures, 273.15K, 283.15K and 298K, were simultaneously fitted using the virial-type [108,109] Equation 1:

$$\ln P = \ln N + \frac{1}{T}\sum_{i=0}^{m} a_i N^i + \sum_{i=0}^{n} b_i N^i \tag{1}$$

The heat of adsorption at zero coverage was calculated from Equation 2, and as a function of surface coverage, from Equation 3:

$$Q_{st} = -Ra_o \tag{2}$$

$$Q_{st}(N) = -R\sum_{i=0}^{m} a_i N^i \tag{3}$$

For the determination of the isosteric heat of adsorption using the Clausius–Clapeyron equation, a commercially available software, ASiQwin (version 3.01), purchased from Quantachrome, was used.

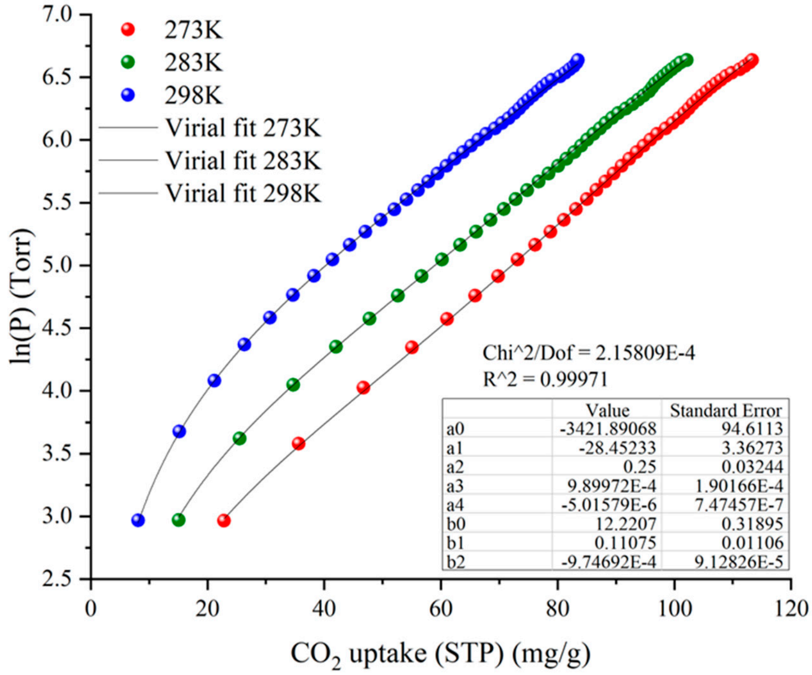

Figure S22. Virial-type fitting of CO_2 adsorption isotherms of compound (**2**)$_n$ at 273K, 283K and 298K according to Equation 1.

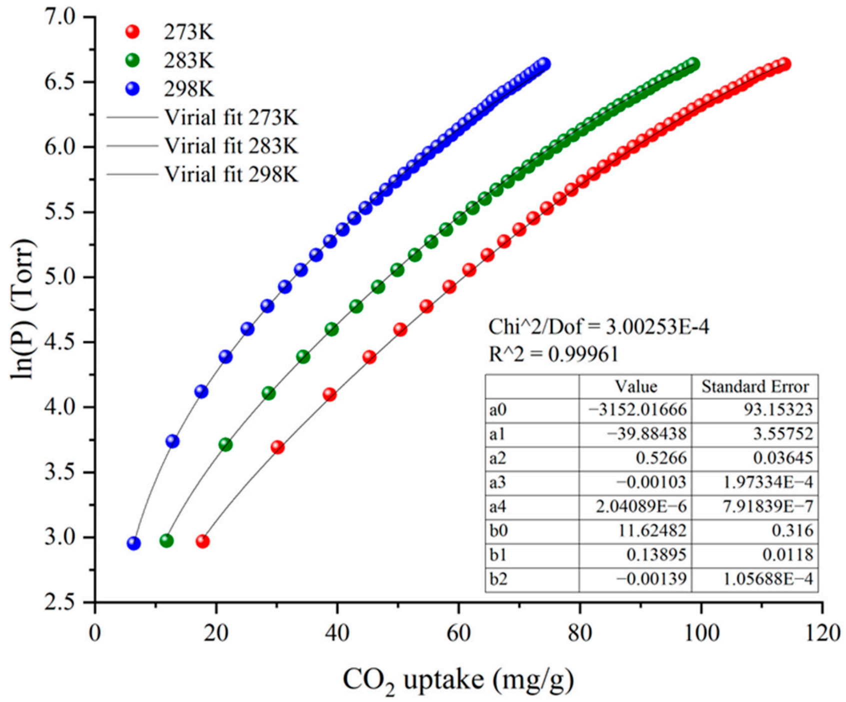

	Value	Standard Error
a0	−3152.01666	93.15323
a1	−39.88438	3.55752
a2	0.5266	0.03645
a3	−0.00103	1.97334E−4
a4	2.04089E−6	7.91839E−7
b0	11.62482	0.316
b1	0.13895	0.0118
b2	−0.00139	1.05688E−4

Figure S23. Virial-type fitting of CO_2 adsorption isotherms of compound (**3**)$_n$ at 273K, 283K, and 298K according to Equation 1.

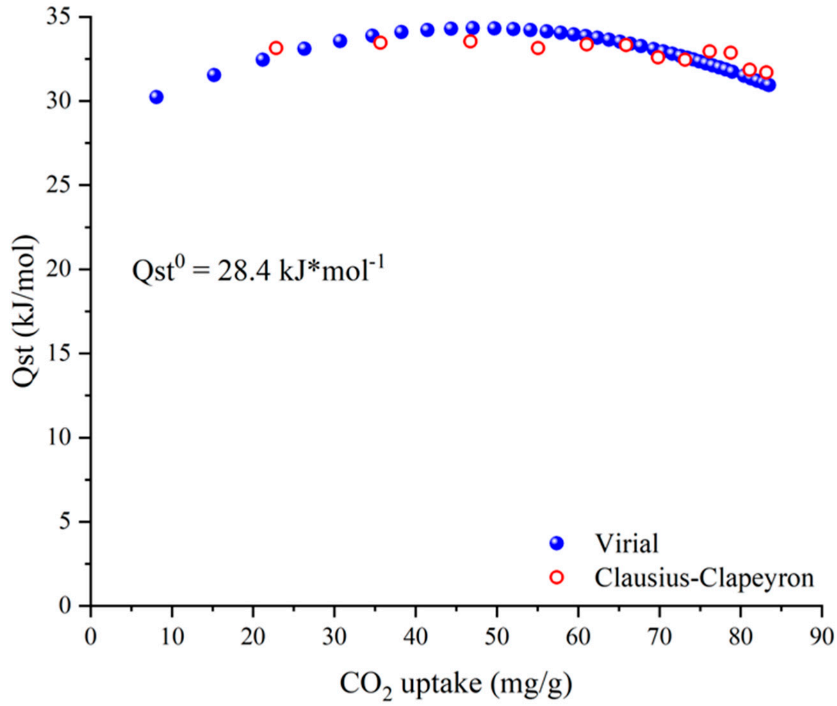

Figure S24. CO_2 isosteric heat of adsorption in compound (**2**)$_n$ as a function of surface coverage.

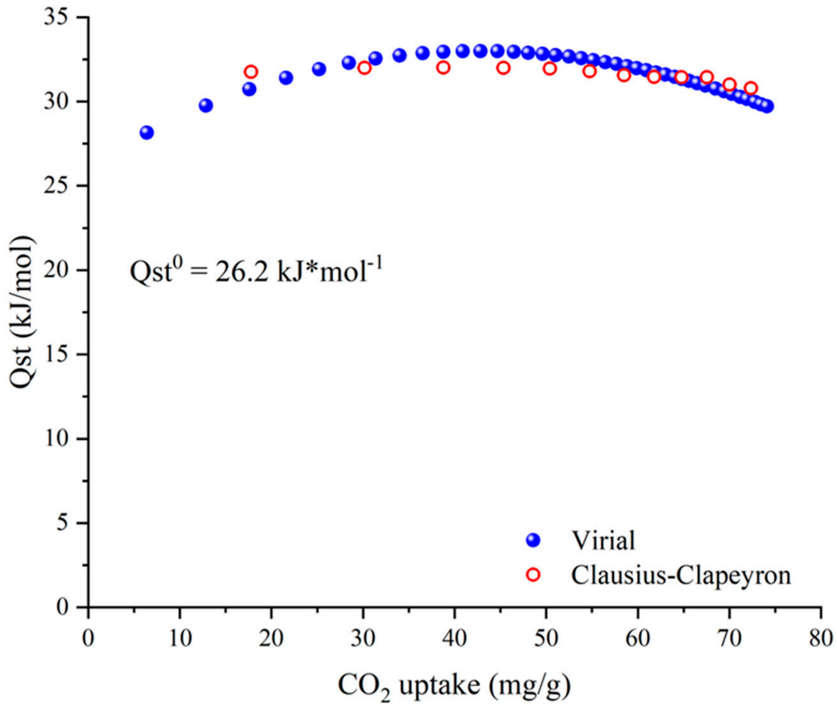

Figure S25. CO_2 isosteric heat of adsorption in compound (**3**)$_n$ as a function of surface coverage.

References

103. Sheldrick, G.M. Crystal Structure Refinement with *SHELXL*. *Acta Crystallogr C Struct Chem* **2015**, *71*, 3–8, doi:10.1107/S2053229614024218.
104. Oxford Diffraction, CrysAlis CCD and CrysAlis RED, Version P171.38.46, Oxford Diffraction Ltd, Abingdon, UK, 2017.
105. Farrugia, L.J. *WinGX* and *ORTEP for Windows* : An Update. *J Appl Crystallogr* **2012**, *45*, 849–854, doi:10.1107/S0021889812029111.
106. Dolomanov, O.V.; Bourhis, L.J.; Gildea, R.J.; Howard, J.A.K.; Puschmann, H. *OLEX2* : A Complete Structure Solution, Refinement and Analysis Program. *J Appl Crystallogr* **2009**, *42*, 339–341, doi:10.1107/S0021889808042726.
107. K. Brandenburg, Version 2003.2001d, Crystal Impact GbR, Bonn, Germany, 2006.
108. CZEPIRSKI, L.; JAGIELLO, J. VIRIAL-TYPE THERMAL EQUATION OF GAS SOLID ADSORPTION. **1988**, *44*, 787–801.
109. Sumida, K.; Rogow, D.L.; Mason, J.A.; McDonald, T.M.; Bloch, E.D.; Herm, Z.R.; Bae, T.-H.; Long, J.R. Carbon Dioxide Capture in Metal–Organic Frameworks. *Chem. Rev.* **2012**, *112*, 724–781, doi:10.1021/cr2003272.
